# Sonic Hedgehog Signaling Is Required for Cyp26 Expression during Embryonic Development

**DOI:** 10.3390/ijms20092275

**Published:** 2019-05-08

**Authors:** Maha El Shahawy, Claes-Göran Reibring, Kristina Hallberg, Cynthia L. Neben, Pauline Marangoni, Brian D. Harfe, Ophir D. Klein, Anders Linde, Amel Gritli-Linde

**Affiliations:** 1 Department of Oral Biochemistry, Sahlgrenska Academy at the University of Gothenburg, SE-40530 Göteborg, Sweden; maha.el.shahawy@odontologi.gu.se (M.E.S.); claes-goran.reibring@gu.se (C.-G.R.); kristina.hallberg@odontologi.gu.se (K.H.); linde@odontologi.gu.se (A.L.); 2 Department of Oral Biology, Minia University, Minia 51161, Egypt; 3 Program in Craniofacial Biology and Department of Orofacial Sciences, University of California San Francisco, San Francisco, CA 94143, USA; cynthianeben@gmail.com (C.L.N.); Pauline.Marangoni@ucsf.edu (P.M.); Ophir.Klein@ucsf.edu (O.D.K.); 4 Department of Molecular Genetics and Microbiology, University of Florida College of Medicine, Gainesville, FL 32610, USA; bharfe@UFL.EDU; 5 Department of Pediatrics and Institute for Human Genetics, University of California San Francisco, San Francisco, CA 94143, USA

**Keywords:** Cyp26 enzymes, congenital anomalies, CRE/LoxP, hedgehog signaling, mouse models, retinoic acid, smoothened, sonic hedgehog

## Abstract

Deciphering how signaling pathways interact during development is necessary for understanding the etiopathogenesis of congenital malformations and disease. In several embryonic structures, components of the Hedgehog and retinoic acid pathways, two potent players in development and disease are expressed and operate in the same or adjacent tissues and cells. Yet whether and, if so, how these pathways interact during organogenesis is, to a large extent, unclear. Using genetic and experimental approaches in the mouse, we show that during development of ontogenetically different organs, including the tail, genital tubercle, and secondary palate, Sonic hedgehog (SHH) loss-of-function causes anomalies phenocopying those induced by enhanced retinoic acid signaling and that SHH is required to prevent supraphysiological activation of retinoic signaling through maintenance and reinforcement of expression of the *Cyp26* genes. Furthermore, in other tissues and organs, disruptions of the Hedgehog or the retinoic acid pathways during development generate similar phenotypes. These findings reveal that rigidly calibrated Hedgehog and retinoic acid activities are required for normal organogenesis and tissue patterning.

## 1. Introduction

Development and homeostasis of multicellular organisms crucially rely on concerted functions of a multitude of proteins and small molecules that operate within signaling pathways. Understanding how signaling pathways interact to ensure normal embryonic development and maintenance of proper shape, size, cellular organization and function of tissues and organs is requisite to decipher the etiopathogenesis of congenital malformations and diseases.

The Hedgehog and retinoic acid (RA) signaling pathways play key roles during embryogenesis, organogenesis, and tissue homeostasis [1,2,3,4,5,6,7,8,9,10,11,12,13], and genetic disruption of Hedgehog signaling can lead to neoplasia [1,14,15,16,17,18]. Mammals produce three Hedgehog ligands, Desert hedgehog, Indian hedgehog (IHH) and Sonic hedgehog (SHH) [1,19]. Hedgehog ligands, notably SHH and IHH proteins, emanating from producing cells, can signal both short and long-range [1,4]. The Hedgehog signaling cascade is regulated by several factors at different levels, from ligand modifications and release to ligand reception and signal transduction [4,6]. In the absence of Hedgehog ligands, the Hedgehog receptor PTCH1 accumulates predominantly in the primary cilium and inhibits Smoothened (SMO), an obligatory factor for the transduction of all Hedgehog signals, leading to the formation of repressor forms of GLI transcription factors that repress Hedgehog target genes. Upon ligand binding to PTCH1, the activated SMO protein translocates to the cilium and initiates a signaling cascade that reaches its acme in the nucleus, where the activator forms of GLI proteins activate transcription of Hedgehog target genes. The principal GLI activator function derives primarily from GLI2, whereas the GLI repressor function largely emanates from GLI3 [1,4,6,18,20,21].

All-trans retinoic acid (RA), the predominant active metabolite of the dietary-derived vitamin A, is a small, highly diffusible and biologically potent lipophilic molecule. During embryonic development, RA is produced from maternally-derived vitamin A. Experimental studies in rodents and avians established the importance of vitamin A for proper development, as vitamin A deficiency during embryogenesis and early organogenesis engenders a wide range of congenital anomalies [22]. However, exposure of embryos to excess vitamin A or RA is teratogenic. Direct evidence for the crucial role of RA during development emanated from genetic gain and loss-of-function studies in mice and zebrafish, which demonstrated that proper tissue patterning and cell fate specification require well-calibrated spatio-temporal RA activity [2,3,5,23,24].

RA synthesis from retinol, the alcohol form of vitamin A, is a stepwise process catalyzed by various dehydrogenases. First, retinol is oxidized into retinaldehyde by alcohol dehydrogenases and retinol dehydrogenases. Thereafter, oxidation of retinaldehyde to RA is catalyzed by retinaldehyde dehydrogeneases, including RALDH1, RALDH2, and RALDH3, encoded by *Aldh1a1*, *Aldh1a2* and *Aldh1a3*, respectively [2,22,24]. RA is degraded by the cytochrome P450 isoenzymes CYP26A1 [25], CYP26B1 [26], and CYP26C1 [27]. Thus, cells expressing CYP26 enzymes are protected from physiological RA activity. RA signaling is mediated by heterodimers of two classes of DNA-binding nuclear receptors that bind to RA response elements (*RARE*) to regulate target gene transcription: (1) the retinoic acid receptors (RARα, RARβ, and RARγ encoded by *RARa*, *RARb* and *RARg*, respectively) which bind to *all-trans* RA and (2) the retinoid X receptors (RXRα, RXRβ, and RXRγ encoded by *RXRa*, *RXRb* and *RXRg*, respectively) that bind to 9-cis-RA. In the absence of ligand, RAR/RXR dimers recruit co-repressors to inhibit transcription of RA target genes, whereas ligand-bound RAR/RXR dimers recruit co-activators to activate the same targets [2,22,24].

Previous studies have shown that cells can respond to both SHH and RA signaling, and that coordinated functions of these pathways are required for normal development. In this respect, during patterning of the spinal cord, SHH and RA exhibit complementary roles in specification of motor neuron progenitor identity [28,29,30]. Likewise, the SHH and RA pathways converge to influence other developmental processes, including patterning and differentiation of the forebrain, early specification of neuronal and mesodermal derivatives, and the establishment of left-right asymmetry [1,31,32,33,34,35,36]. RA and Hedgehog activities may also directly control expression of the same target genes, as exemplified by the existence of functional GLI and RAR-RXR binding sites in the *Ngn2* enhancer [37]. However, in other biological settings SHH has been shown to oppose RA activity. In the developing limb for example, SHH operates within a signaling network to promote proximal-distal growth by enhancing CYP26B1-mediated RA degradation [38]. In the human bone marrow, multiple myeloma cells modify their microenvironment to escape differentiation and reinforce chemoprotection by inhibiting RA activity in the stroma through SHH-mediated upregulation of *CYP26A1* expression [39].

Recently, we showed that in the developing tongue antagonistic activities of SHH and RA control patterning, growth and epithelial cell fate specification and that SHH inhibits RA inputs through maintenance and enhancement of *Cyp26a1* and *Cyp26c1* expression in the lingual epithelium [40]. While reviewing the literature pertaining to the RA and Hedgehog signaling pathways, we noticed that in several tissues and organs loss of Hedgehog signaling generates malformations that are strikingly similar to those engendered by supraphysiological activation of RA signaling. We therefore sought to determine whether in murine tissues known to depend on SHH for normal development, SHH antagonizes RA signaling through CYP26. To this end, we used mutant mice lacking SHH signaling and complementary experimental approaches in vitro. We found that loss of SHH signaling causes indeed loss of expression of *Cyp26* genes and enhancement of RA signaling during ontogeny of organs as disparate as craniofacial structures, genital tubercle and tail, and generates anomalies mimicking those engendered by genetically or pharmacologically induced activation of RA signaling. These findings show that in different developing organs SHH signaling uses a common strategy to antagonize RA activity. Our findings provide a concept to further the understanding of the pathogenesis of congenital malformations caused by altered Hedgehog signaling and the mechanisms underlying Hedgehog-dependent tumorigenesis.

## 2. Results and Discussion

To determine whether, as in the developing tongue [40], SHH signaling also impinges upon RA activity in other embryonic structures, we generated and studied *K14-CRE/Shh^f/f^* mutant embryos, in which the *Shh* gene is disabled in Keratin-14 expressing cells and their progeny [40,41], as well as *ShhGFPCRE/Smo^f/f^* and *ShhCreER^T2^/Shh^f^* mutant embryos, which lack the function of the *Smo* and *Shh* genes, respectively, in cells that express *Shh* and their progeny [40,41,42,43]. In the *ShhGFPCRE/Smo^f/f^* mutants, only cells that express or have expressed SHH are unable to respond to SHH signaling. In the *ShhCreER^T2^/Shh^f^* mutants exposure to tamoxifen (TAM) abrogates SHH production, leading to loss of both autocrine and paracrine SHH signaling. Similary, in the *K14-CRE/Shh^f/f^* mutants, both autocrine and paracrine SHH signaling are disabled. Embryos not expressing the CRE gene and/or the floxed *Smo* and *Shh* alleles were phenotypically normal; they were thus used as controls [40,41,42].

### 2.1. SHH Signaling Antagonizes RA Activity through CYP26A1 to Ensure Proper Development of the Tail

Experimental and genetic studies have demonstrated that SHH emanating from the notochord, a mesodermal midline rod-like structure, and the neural floor plate is required for survival and expansion of the sclerotomes, somite-derived structures that form the vertebral column [1,44]. Homozygous *Shh* null (*Shh^n/n^*) mutant embryos, in which *Shh* is disabled in the germ line exhibit severe axial defects with nearly total absence of sclerotomal derivatives, including the entire vertebral column [44]. In the *Shh^n/n^* mutants, the notochord differentiates, but is subsequently lost, indicating that autocrine SHH signaling is essential for maintenance of this important structure [44]. After fulfilling its function in patterning adjacent tissues, the notochord persists only in prospective intervertebral discs, where it develops into the *nucleus pulposus*. *ShhGFPCRE/Smo^f/f^* and TAM-induced *ShhCreER^T2^/Shh^f^* mutants, in which abrogation of SHH signaling occurs shortly after formation of the notochord and floor plate, exhibit an abnormally thin notochord and lack intervertebral discs in the thoracic and lumbar regions. The latter anomaly is due to loss of notochordal integrity, leading to failure of development of the *nucleus pulposus* [42].

**Figure 1:**
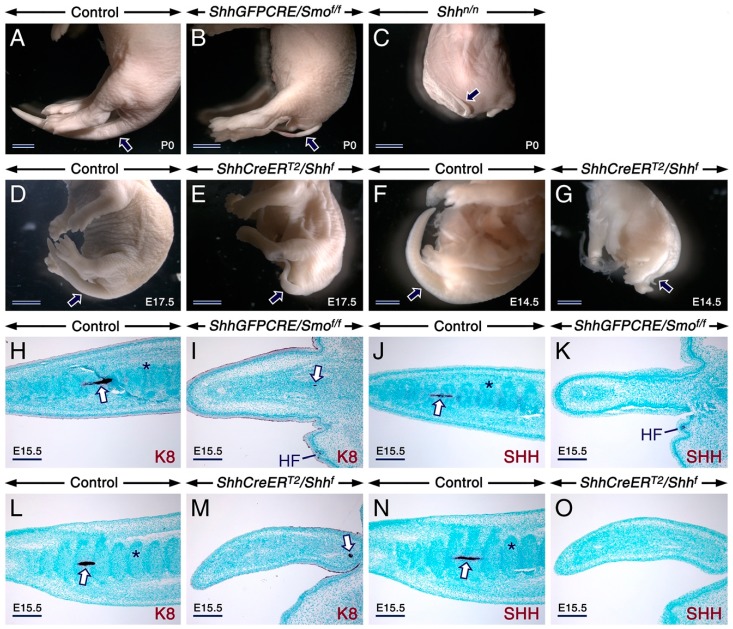
Loss of sonic hedgehog (SHH) signaling generates an abnormally thin and truncated tail lacking the notochord and vertebral chondrogenic condensations. (**A**–**G**). Representative external tail phenotype (arrows) of mutants relative to controls. Control (**A**; *n* = 15), *ShhGFPCRE/Smo^f/f^* mutant (**B**; *n* = 11), and *Shh^n/n^* mutant (**C**; *n* = 2) newborns (P0). E17.5 control (**D**; *n* = 8) and *ShhCreER^T2^/Shh^f^* mutant (**E**; *n* = 9) embryos first exposed to tamoxifen (TAM) at E11.5. E14.5 control (**F**; *n* = 5) and *ShhCreER^T2^/Shh^f^* mutant (**G**; *n* = 6) embryos first exposed to TAM at E10.5. The mutants exhibit severe tail defects. (**H**–**O**) Tail sections from E15.5 mutants and controls immunostained (dark purple) for Keratin 8 (K8) and Sonic hedgehog (SHH) to visualize the notochord. Tails from a control embryo (**H**,**J**) and a *ShhGFPCRE/Smo^f/f^* embryo (**I**,**K**). Tails from a control embryo (**L**,**N**) and a *ShhCreER^T2^/Shh^f^* mutant embryo (**M**,**O**) first exposed to TAM at E10.5. The control tails display chondrogenic mesenchymal condensations of presumptive vertebrae (asterisks) and a notochord (arrows) in the caudal region, whereas the mutant tails lack these structures. K8-positive (arrows in **I** and **M**) remnants of the notochord are visible in the rostral region of the mutant tails. HF, hair follicle. Scale bars: 2 mm (**A**–**C**), 1 mm (**D**–**G**) and 200 µm (**H**–**O**).

*Shh^n/n^*, *ShhGFPCRE/Smo^f/f^* and TAM-induced *ShhCreER^T2^/Shh^f^* mutants all display a severely truncated and abnormally thin tail totally lacking vertebrae [42,44] (see also Figure 1A–G). Furthermore, immunostaining for SHH and Keratin 8, molecular markers of the notochord and *nucleus pulposus* [42,45,46], showed that in contrast to control tails which exhibited a notochord, the mutants tails were devoid of this structure, except rostrally, where an abnormally thin Keratin 8-positive notochord was detectable (Figure 1H–O). Development of vertebrae is heralded by condensation of sclerotome-derived chondrogenic mesenchymal cells. These structures failed to develop in the mutant tails (Figure 1H–O), consistent with failure of development of tail vertebrae upon loss of SHH signaling [42,44].

Tail development initiates in the future lumbosacral region and coincides with the closure of the posterior neuropore. Tail tissues, including the neural tube, notochord and somites, originate from the tail bud mesenchyme, a progenitor zone located at the tip of the embryonic tail. The hindgut extends a short distance into the elongating tail after closure of the posterior neuropore [47]. The developing tail expresses components of the SHH and RA pathways. SHH is produced in the notochord and neural floor plate and elicits responses in the notochord, neuroepithelium, as well as in somites and sclerotomes [1,42]. *Aldh1a2* is expressed in presomitic and somitic mesoderm anterior to the tail bud [22,48,49], whereas *RARs* are expressed in presomitic and somitic mesoderm, sclerotomes, and tail bud [22,47,50,51,52]. RA signaling is tightly controlled by the activities of RALDHs and CYP26s, and loss-of-function of CYP26s during development leads to supraphysiological activation of RA signaling with entailing congenital malformations [53,54]. In the embryonic tail, *Cyp26a1* is expressed at high levels in the tail bud mesoderm, the neuroepithelium and hindgut endoderm [25,55,56,57,58].

Remarkably, the tail phenotype characterized by formation of a truncated and thin tail lacking vertebrae in the *Shh^n/n^*, *ShhGFPCRE/Smo^f/f^* and TAM-induced *ShhCreER^T2^/Shh^f^* mutants is strikingly similar to that in *Cyp26a1^n/n^* mice [25,59,60] and rodent embryos exposed to teratogenic doses of vitamin A or RA [61,62,63,64,65]. Furthermore, exposure of hamster embryos to exogenous RA causes degeneration of the notochord and alters the formation of axial chondrogenic condensations [66], mimicking the anomalies caused by loss of SHH signaling. *Cyp26b1* and *Cyp26c1* are not expressed during the critical, SHH-dependent stages of tail formation [57,67] and embryos with loss-of-function of *Cyp26b1* [26,68] and *Cyp26c1* [27] do not exhibit tail truncation. *Cyp26b1* transcripts become detectable at later developmental stages concomitantly with the formation of chondrogenic mesenchymal condensations prefiguring vertebrae [69]. These become visible in the proximal part of the caudal region of mouse embryos at E12.5-E13 [70]. It is noteworthy that chondrogenic mesenchymal condensations express *Indian Hedgehog* [71,72]. These observations may be taken to suggest that the tail defects engendered by loss of SHH signaling are caused, at least in part, by abnormal activation of RA signaling owing to loss CYP26A1-mediated RA degradation.

**Figure 2:**
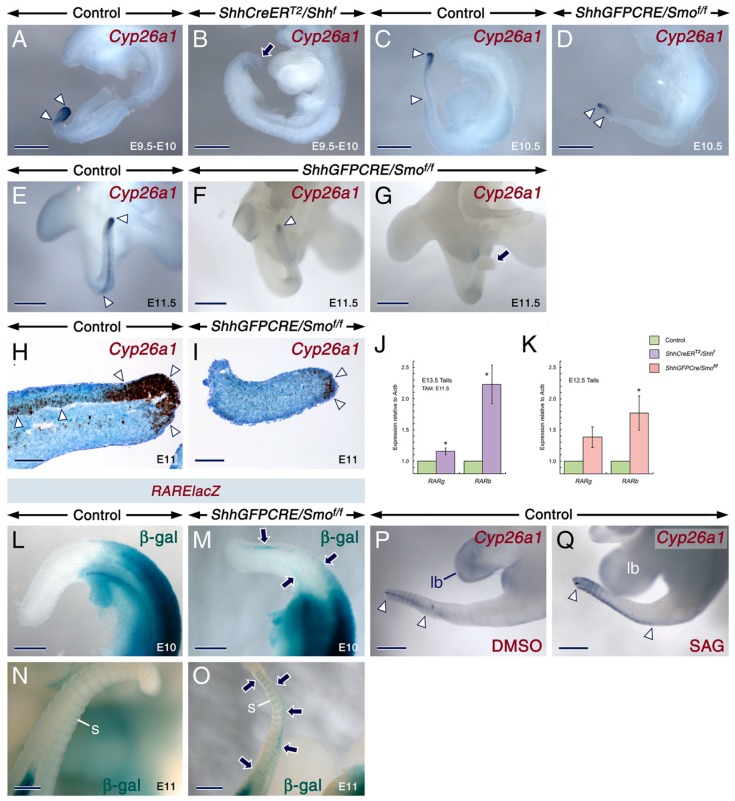
Loss of SHH signaling in the developing tail causes loss of *Cyp26a1* expression and ectopic activation of retinoic acid signaling. (**A**–**G**) Representative whole-mount in situ hybridization (ISH) with riboprobes showing *Cyp26a1* expression (purple) in developing tails. E9.5-E10 control (**A**; *n* = 3) and *ShhCreER^T2^/Shh^f^* mutant (**B**; *n* = 4) embryos first exposed to tamoxifen (TAM) at E8-E8.5. Control (**C**,**E**) and *ShhGFPCRE/Smo^f/f^* mutant (**D**,**F**,**G**) embryos at E10.5 (**C**,**D**; *n* = 4 controls and *n* = 4 mutants) and E11.5 (**E**–**G**; *n* = 4 controls and *n* = 3 mutants). In the control tails, the *Cyp26a1* expression domain extends from the tail bud to more rostral levels of the tail (arrowheads in **A**,**C** and **E**). The mutant tails exhibit either a severely reduced domain of *Cyp26a1* expression (arrowheads in **D** and **F**) or abolished *Cyp26a1* expression (arrows in **B** and **G**). (**H**,**I**) Representative tail sections from E11 control embryos (**H**; *n* = 2) and a *ShhGFPCRE/Smo^f/f^* mutant embryo (**I**) after ISH for *Cyp26a1* with oligonucleotide probes (black). Decreased *Cyp26a* hybridization signals in the mutant tail as compared to the control tail (arrowheads in **H** and **I**). (**J**,**K**) RT-qPCR analysis showing the expression levels of *RARb* and *RARg* relative to *Actb* (β-actin). Upregulation of *RARb* (*p* = 0.0162) and *RARg* (*p* = 0.0261) levels in tails from E13.5 *ShhCreER^T2^/Shh^f^* mutant (*n* = 3 and *n* = 4 for *RARb* and *RARg* analyses, respectively) as compared to tails from control (*n* = 3 and *n* = 4 for *RARb* and *RARb* analyses, respectively) embryos first exposed to TAM at E11.5 (**J**). Upregulation of *RARb* (*p* = 0.0476) and *RARg* (*p* = 0.0610) levels in tails from E12.5 *ShhGFPCRE/Smo^f/f^* mutants (*n* = 3 and *n* = 4 for *RARb* and *RARg* analyses, respectively) as compared to tails from controls (*n* = 3 and *n* = 4 for *RARb* and *RARg* analyses, respectively) (**K**). Data are mean values ± standard deviation; *: *p* < 0.05. (**L**–**O**) Representative β-galactosidase (β-gal) histochemistry visualizing retinoic acid activity (blue) in control (**L**,**N**) and *ShhGFPCRE/Smo^f/f^* mutant (**M**,**O**) embryos carrying the *RAREhsplacZ* transgene (*RARElacZ*) at E10 (**L**,**M**; *n* = 3 controls and *n* = 3 mutants) and at E11 (**N**,**O**; *n* = 7 controls and *n* = 3 mutants). The mutants exhibit ectopic retinoic acid activity (arrows in **M** and **O**) in tail tissues. s, somite. (**P**,**Q**) Representative tail explants from E11.5 control embryos treated for 24 h with DMSO (**P**; *n* = 5) and 0.2 µM SAG (**Q**; *n* = 4) showing expansion of *Cyp26a1* expression domain (arrowheads in **P** and **Q**) and increased *Cyp26a1* hybridization signals in the SAG-treated tail and failure of SAG to induce ectopic *Cyp26a1* expression in adjacent structures, including the hindlimb bud (lb). Scale bars: 300 µm (**A**–**G**,**L**–**Q**) and 100 µm (**H**,**I**).

To explore this possibility, we assessed the expression levels of *RARb* and *RARg*, well-established direct transcriptional targets of RA signaling [23], as well as the expression patterns of *Cyp26a1* in control and mutant tails. Reverse transcription quantitative PCR (RT-qPCR) revealed significant upregulation of *RARb* and *RARg* transcripts in tails from *ShhGFPCRE/Smo^f/f^* and TAM-induced *ShhCreER^T2^/Shh^f^* mutants (Figure 2J,K). Furthermore, *Cyp26a1* in situ hybridization signals were either abolished or dramatically diminished in the mutant tails (Figure 2A–I).

RA activity can be visualized in tissues from mice carrying the *RAREhsplacZ* transgene [73]. Although this transgene fails to accurately reveal RA activity in several tissues and organs, including the developing tongue [40,73,74,75,76] and a large part of the palatal shelves of the secondary palate [77], it is able to visualize abnormal activation of RA signaling in the developing tail [59,60]. We thus took advantage of this possibility by examining tails from controls and *ShhGFPCRE/Smo^f/f^* mutants carrying the *RAREhsplacZ* transgene and found that similar to *Cyp26a1^n/n^* embryos [60] the *ShhGFPCRE/Smo^f/f^* mutants exhibited expansion of *RAREhsplacZ* activity in the developing tail (Figure 2L–O), indicating ectopic activation of RA signaling. Taken together, these findings show that loss of SHH signaling in the developing tail causes a decrease of *Cyp26a1* expression and enhancement of RA signaling.

Recently, we showed that in the developing tongue, SHH activity is required for maintenance and reinforcement of *Cyp26a1* and *Cyp26c1* expression but not for the initiation of their expression [40]. This phenomenon occurs also in the developing tail, since in vitro treatment of tails with SAG, a SMO agonist enhanced the intensity of *Cyp26a1* hybridization signals in tails but failed to induce ectopic *Cyp26a1* expression in adjacent tissues (Figure 2P,Q).

**Figure 3:**
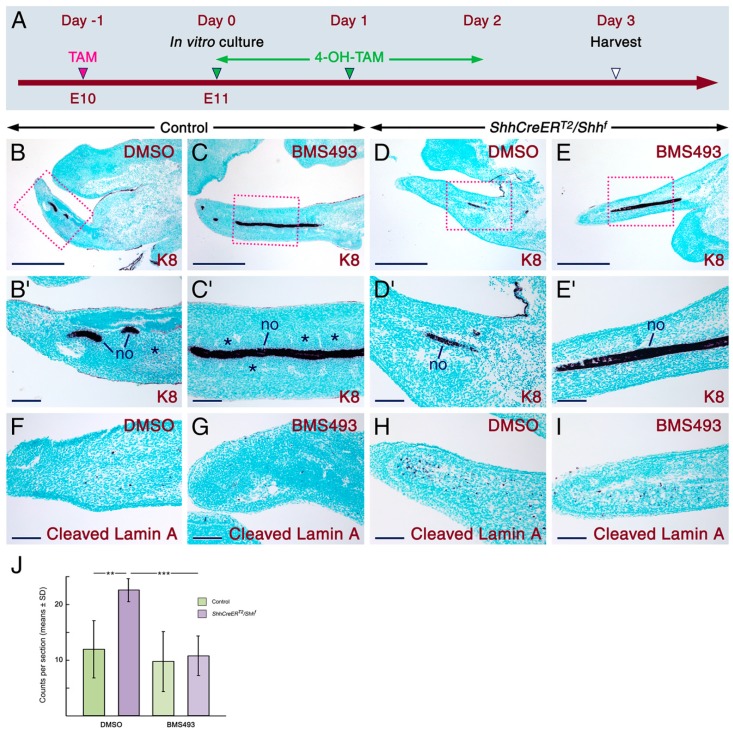
In vitro inhibition of retinoic acid signaling partially rescues the tail phenotype of SHH-deficient embryos. (**A**) Timeline representing the induction of CRE-mediated deactivation of *Shh* in embryos and in tail explants. The tails are from E11 control and *ShhCreER^T2^/Shh^f^* mutant embryos first exposed in utero to tamoxifen (TAM) at E10 (red arrowhead). All tail explants were cultivated in vitro for two days in the presence of 4-hydroxytamoxifen (4-OH-TAM; green arrowheads). During the in vitro cultivation period (three days), the tails were treated with DMSO or 12.5 µM BMS493. The time of harvest of the explants is indicated by a white arrowhead. (**B**–**E**) Representative Keratin 8 (K8; dark purple) immunostaining visualizing the notochord (no) in sections of tail explants from control and mutant embryos. The tails were treated with DMSO (*n* = 5 controls and *n* = 6 mutants) or BMS493 (*n* = 13 controls and *n* = 8 mutants). **B’**–**E’** are magnified images of the boxed areas in **B**–**E**. All the control tails treated with DMSO (**B**,**B’**) or BMS493 (**C**,**C’**) exhibit a notochord and chondrogenic mesenchymal condensations (asterisks in **B’** and **C’**). All the DMSO-treated mutant tails lack a notochord in the posterior region, while in the rostral region they display an abnormally thin notochord (**D**,**D’**). The BMS493-treated mutant tails (**E**,**E’**) display a notochord (*n* = 6/8), but fail to exhibit chondrogenic mesenchymal condensations (*n* = 8/8). (**F**–**I**) Representative sections of tail explants from control and mutant embryos were immunostained for cleaved Lamin A (dark purple) to visualize apoptotic cells. Massive apoptosis in the DMSO-treated mutant tails (**H**; *n* = 6) as compared to the BMS493-treated mutant tails (**I**; *n* = 6) and the DMSO-treated (**F**; *n* = 3) and BMS493-treated (**G**; *n* = 7) control tails. (**J**) Quantitation of apoptosis in tail explants (the number of explants assessed is described above). The number of apoptotic cells in the DMSO-treated mutant tails is significantly higher than in the DMSO-treated (*p* < 0.005) and BMS493-treated (*p* = 0.002) control tails. The BMS493-treated mutant tails show a significant decrease in apoptosis, as compared to the DMSO-treated mutant tails (*p* < 0.001). BMS493 had no effects on the extent of apoptosis in the control tails (*p* = 0.59). Data are mean values ± standard deviation; **: *p* < 0.01; ***: *p* < 0.001. Scale bars: 500 µm (**B**–**E**) and 100 µm (**B’**–**I**).

To determine whether increased RA signaling is indeed involved in the genesis of tail anomalies upon loss of SHH signaling, we cultured tails from TAM-treated *ShhCreER^T2^/Shh^f^* mutant and control embryos (Figure 3A) in the presence of BMS493, a RA signaling inhibitor, or DMSO (control vehicle). Compared to tails from control embryos, the DMSO-treated mutant tails exhibited an abnormally thin notochord in the rostral region and were devoid of notochord in the posterior region (Figure 3B–3D’). However, the BMS493-treated mutant tails exhibited an intact notochord (Figure 3E,E’), indicating that degeneration of the notochord was prevented upon inhibition of RA signaling. These data suggest that RA signaling participates in the degeneration of the caudal notochord upon loss of SHH signaling. However, compared to tails from control embryos treated with DMSO or BMS493 (Figure 3B–C’), the BMS493-treated mutant tails failed to show chondrogenic mesenchymal condensations flanking the notochord (Figure 3D,D’), indicating that the inhibition of RA signaling only partially rescued the mutant tails. This finding was not surprising, as survival and expansion of sclerotomal cells, which form axial chondrogenic condensations, are SHH-dependent [1,44].

RA activity is required for apoptosis-mediated removal of the interdigital mesenchyme [78], and genetic or teratogenic overactivation of RA signaling is known to induce apoptosis in developing organs, including the testes, limb mesenchyme, chondrogenic mesenchymal condensations [54], and the developing tail [65,79]. Loss of SHH signaling in the developing tail causes enhanced apoptosis [42]. Accordingly, the TAM-induced *ShhCreER^T2^/Shh^f^* mutant tails treated with DMSO exhibited increased numbers of apoptotic cells, as compared to the DMSO-treated tails from control embryos (Figure 3F,H,J). We also found that BMS493 treatment significantly reduced the number of apoptotic cells in the mutant tails (Figure 3H–J). These findings strongly suggest that enhanced apoptosis in the mutant tails is at least partly caused by ectopic activation of RA signaling.

Altogether, our data reveal a hitherto unknown mechanism behind abnormal tail development upon loss of SHH signaling and strongly suggest involvement of ectopic RA activation in the genesis of this anomaly. The fact that loss of SHH signaling [42,44] (this study) and ectopic activation of RA signaling [25,59,60,61,62,63,64,65] during tail development generates strikingly similar tail defects further supports our conclusion.

### 2.2. SHH Signaling in the Developing Secondary Palate Is Required to Prevent Enhancement of RA Activity

Development of the secondary palate depends on complex spatio-temporal cellular and molecular events, and genetic mutations and/or environmental factors that alter these events cause cleft palate, the most common congenital malformation in humans, with debilitating consequences [80,81,82,83,84]. In mice, development of the secondary palate begins at E11.5. First, bilateral paired palatal shelves (PS) arise from the oral side of the maxillary processes and grow downwards while flanking the developing tongue (E11.5-E14.5). Thereafter, the PS elevate to a horizontal position (E14.5-E15) above the tongue, and further growth enables the opposing PS to adhere to each other and form a median epithelial seam which eventually disappears, allowing fusion of the PS [80,81,82,83,84]. The developing palate exhibits molecular and histological heterogeneity along its anterior-posterior and oral-nasal axes. Along the oral-nasal axis, this heterogeneity is translated into formation of ciliated respiratory epithelium that differentiates on the nasal side of the PS, and development of periodic epithelial ridges known as *rugae palatinae* in the oral surface of the PS [80,84].

SHH signaling plays a crucial role during PS growth and patterning of *rugae palatinae* through regulation of the expression of signaling molecules and transcription factors, and loss of SHH signaling causes cleft palate [6,80,84,85,86] and mispatterning of palatal *rugae* [86]. During palatogenesis, SHH is produced by the PS epithelium and signals within the PS epithelium and to the PS mesenchyme. At E11.5 *Shh* is expressed in the entire epithelium of the emerging PS, and from E12 onwards *Shh* expression is restricted to the developing *rugae palatinae* [86,87,88,89,90,91].

In the developing palate, *RARs* are expressed in both the epithelium and mesenchyme [92], whereas, *Cyp26a1* [56] and *Cyp26b1* [69,77] are expressed in the epithelium and mesenchyme, respectively. Interestingly, *Cyp26a1* expression is restricted to the oral epithelium of the PS [56], overlapping with *Shh* expression [86,87,88,89,90,91]. Exposure of rodent embryos to excess RA or vitamin A causes cleft palate [61,62,63,64,93], and *Cyp26b1^n/n^* mice exhibit cleft palate [68,77] due to failure of elevation of PS [77].

**Figure 4:**
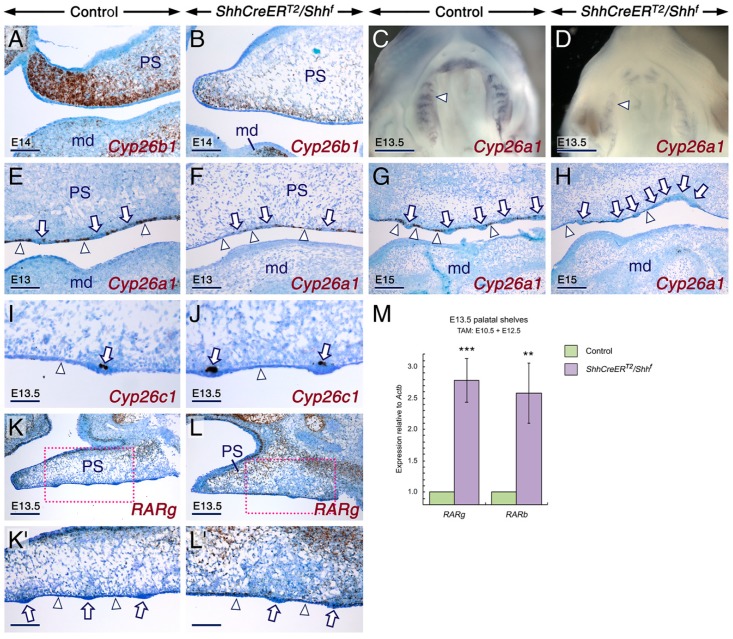
SHH signaling in the developing secondary palate is required for expression of *Cyp26a1* and *Cyp26b1* to prevent enhancement of retinoic acid signaling. (**A**–**L**) Representative developing palates from control and *ShhCreER^T2^/Shh^f^* mutant embryos first exposed to tamoxifen (TAM) at E10-5-E11. The developmental stages are indicated on the panels. Whole-mount in situ hybridization (WMISH) with Dig-labelled riboprobes (**C**,**D**) and in situ hybridization in parasagittal sections (anterior palatal region towards the left of the panels) with oligonucleotide probes (**A**,**B**,**E**–**L**). The inter-rugal epithelium and *rugae palatinae* are indicated by arrowheads and arrows, respectively. (**A**,**B**) *Cyp26b1* expression in sections of palates (see also Figure S4) from control (**A**, *n* = 2) and mutant (**B**; *n* = 2) embryos. The mutant palate shows decreased *Cyp26b1* hybridization signals (brown) as compared to the control palate. (**C**–**H**) The mutant palates (**D**,**F**,**H**; *n* = 3 for WMISH and *n* = 4 for ISH in sections) show decreased *Cyp26a1* hybridization signals (dark purple in whole-mounts and black in sections) as compared to control palates (**C**,**E**,**G**; *n* = 3 for WMISH and *n* = 4 for ISH in sections). In control palates *Cyp26a1* transcripts are enriched in the inter-rugal epithelium. (**I**,**J**) *Cyp26c1* (black) is expressed in subsets of cells within the basal layer of *rugae palatinae* (arrows in **I** and **J**) in control (**I**; *n* = 2) and mutant (**J**; *n* = 2) palates. (K,L) The mutant palate (**L**; *n* = 3) shows increased *RARg* hybridization signals (brown) in the mesenchyme and inter-rugal epithelium as compared to the control palate (**K**; *n* = 3). **K’** and **L’** are magnified views of the boxed areas in **K** and **L**, respectively. (**M**) RT-qPCR assay for *RARb* and *RARg* relative to *Actb* (β-actin) in paired palatal shelves from E13.5 controls (*n* = 7) and *ShhCreER^T2^/Shh^f^* mutants (*n* = 7) first exposed to TAM at E10.5 showing upregulation of *RARb* (*p* = 0.004) and *RARg* (*p* = 0.000) in the mutant palatal shelves as compared to the control palatal shelves. Data are mean values ± standard deviation; **: *p* < 0.01; ***: *p* < 0.001. md, mandible; PS, palatal shelf. Scale bars: 500 µm (**C**,**D**), 200 µm (**K**,**L**), 100 µm (**A**,**B**,**E**–**H**,**K’**,**L’**) and 50 µm (**I**,**J**).

To determine whether ablation of SHH signaling causes enhancement of RA signaling during palate development, we generated *ShhCreER^T2^/Shh^f^* mutant and control embryos that had been first exposed to TAM at E10.5-E11 (Figure S1). We found that all the E10.5-E11 TAM-induced *ShhCreER^T2^/Shh^f^* mutants assessed displayed cleft palate, a defect that was not observed in control embryos (Figure S2A–F). RT-qPCR revealed that *RARg* and *RARb* expression levels were significantly enhanced in the mutant PS (Figure 4M), indicating enhanced RA activity.

To explore putative sources of RA in the developing palate, we assessed the expression patterns of RALDH1-3 proteins in TAM-treated control and *ShhCreER^T2^/Shh^f^* mutant embryos. We found that all three RALDHs were expressed in the developing palate at E13.5 and that their expression patterns were not altered in the mutant palate (Figure S3). These findings show that RA synthesis occurs in the developing palate and that enhanced RA signaling in the *ShhCreER^T2^/Shh^f^* mutant palate is not caused by increased RA synthesis. Interestingly, *Cyp26b1* loss-of-function generates cleft palate owing to enhanced RA signaling and abnormal mesenchymal proliferation in the bend region of the PS [77], a site that we found to be enriched in RALDH1-3 expression (Figure S3).

To determine whether increased RA signaling in the *ShhCreER^T2^/Shh^f^* mutant palate is due to decreased CYP26-mediated RA catabolism, we assessed the expression patterns of *Cyp26a1*, *Cyp26b1*, and *Cyp26c1* transcripts in the palate of TAM-treated control and *ShhCreER^T2^/Shh^f^* mutant embryos. In both control and mutant embryos, *Cyp26b1* displayed a gradient of hybridization signals along the anterior-posterior axis of the PS, with highest and lowest intensities seen anteriorly and posteriorly, respectively (Figure 4A,B and Figure S4). However, compared to control PS, the *ShhCreER^T2^/Shh^f^* mutant PS showed diminished *Cyp26b1* hybridization signals (Figure 4 and Figure S4). Furthermore, *Cyp26a1* hybridization signals were diminished in the epithelium of the mutant PS (Figure 4C–H). By contrast, *Cyp26c1* expression, which we found to be restricted to subsets of cells in *rugae palatinae*, was unaltered in the mutant PS (Figure 4I,J). Notably, in the control PS, *Cyp26a1* transcripts were enriched in the inter-rugal epithelium (Figure 4C,E,G). Consistent with *RARg* RT-qPCR analysis (Figure 4M), the mutant PS displayed increased *RARg* hybridization signals in the palatal mesenchyme and in the inter-rugal epithelium (Figure 4K–L’). Thus, loss of SHH signaling in the developing palate leads to enhanced RA signaling in both the palatal epithelium and palatal mesenchyme as a result of loss of *Cyp26a1* and *Cyp26b1* expression.

**Figure 5:**
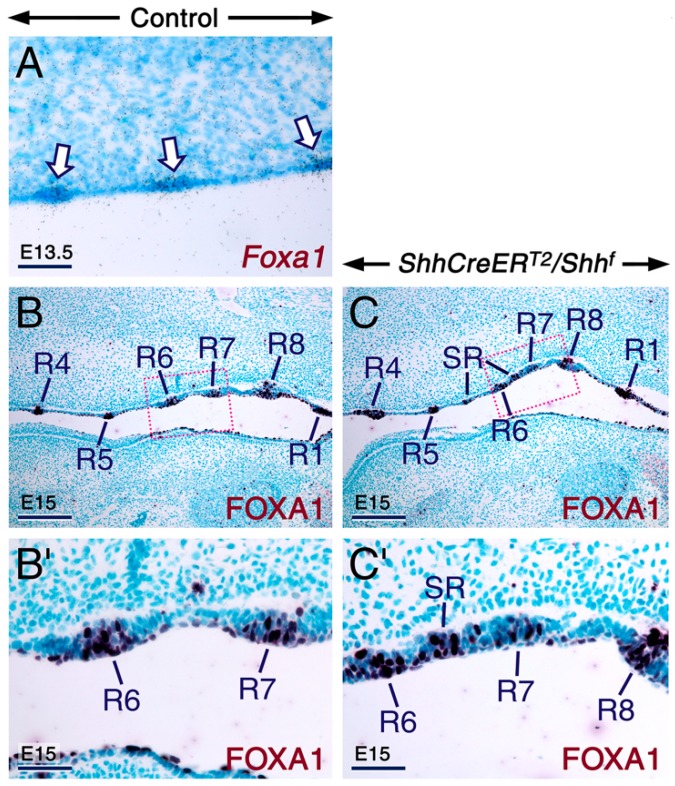
Loss of SHH signaling in the *ShhCreER^T2^/Shh^f^* mutant palate causes mispatterning of *rugae palatinae* (**A**) Bright-field view of a frontal section across the palate of an E13.5 control embryo after in situ hybridization with a ^35^S-UTP-labelled *Foxa1* riboprobe showing *Foxa1* expression (black dots) in *rugae palatinae* (arrows). (**B**,**C**) Representative FOXA1 immunostaining (dark purple) of para-sagittal sections of palates (anterior palatal region towards the left of the panels) from E15 control (**B**; *n* = 3) and *ShhCreER^T2^/Shh^f^* mutant (**C**; *n* = 3) embryos first exposed to tamoxifen at E10.5-E11. **B’** and **C’** are magnified views of the boxed areas in **B** and **C**, respectively. In the control palate FOXA1 is detected in the palatal periderm and in a subset of cells of *rugae palatinae*. The orthotopic *rugae* (R) are labelled with arabic numerals according to the order of their formation as described previously [91]. In the mutant palate supernumerary *rugae* (SR) develop between *rugae* R5 and R6 and between *rugae* R6 and R7 (**C**,**D**). Scale bars: 200 µm (**B**,**C**) and 50 µm (**A**,**B’**,**C’**).

Abrogation of SHH signaling in *K14-Cre/Shh^f/f^* mutant mice causes mispatterning of *rugae palatinae* manifested as furcations, fusions, and formation of supernumerary *rugae* [86] similar to those observed in rat embryos exposed to excess RA [94]. To determine whether, like the *K14-Cre/Shh^f/f^* mutants, the TAM-induced *ShhCreER^T2^/Shh^f^* embryos exhibit mispatterning of *rugae palatinae*, we immunostained sections of control and *ShhCreER^T2^/Shh^f^* mutant palates for FOXA1 whose encoding gene is expressed in *rugae palatinae* [95] (see also Figure 5A). In the oral epithelium of control palates, FOXA1 was expressed in the periderm and *rugae palatinae* (Figure 5B,B’). However, in the *ShhCreER^T2^/Shh^f^* mutant palates, FOXA1 expression was expanded (Figure 5C,C’), indicating development of supernumerary *rugae*. Taken together, these findings show that loss of SHH signaling during palatogenesis leads to enhanced RA signaling and suggest involvement of enhanced RA signaling in the genesis of cleft palate and mispatterning of *rugae palatinae* upon loss of SHH inputs in the developing palate.

Our study revealed a new function for SHH signaling during growth of the PS, which is to keep RA activity in check in both the palatal epithelium and palatal mesenchyme. It is possible that elevated RA availability in the palatal epithelium of the TAM-induced *ShhCreER^T2^/Shh^f^* mutant embryos (as a result of diminished RA degradation by CYP26A1) not only causes enhanced RA signaling within the palatal epithelium, but also contributes in enhancing RA signaling in the palatal mesenchyme, since RA is a highly potent and diffusible small molecule. Vice versa, in the TAM-induced *ShhCreER^T2^/Shh^f^* mutant palates, RA overproduced in the palatal mesenchyme (as a result of diminished RA degradation by CYP26B1) may also contribute to enhancement of RA signaling in the palatal epithelium.

Nature is replete with repeating, regularly spaced structures such as *rugae palatinae*, feather and hair follicles, lingual fungiform papillae, and tracheal cartilage rings. We have shown recently that antagonistic SHH and RA activities are involved in patterning of the lingual epithelium, whereby SHH inhibits while RA promotes the formation of taste placodes and lingual glands [40]. Patterning of *rugae palatinae* has been shown to involve Turing-based mechanisms, where FGF and SHH function as activator and inhibitor, respectively [86]. However, how SHH inhibits *rugae* formation is unknown. In addition, besides the SHH-FGF signaling pair, other signaling pathways have been incriminated in patterning of *rugae palatinae* [86]. In the present study, we confirmed that SHH inhibits the formation of *rugae palatinae*, since in the TAM-induced *ShhCreER^T2^/Shh^f^* mutants the palate forms supernumerary *rugae*. Furthermore, our findings suggest that RA signaling is involved in patterning of *rugae palatinae*. Several lines of evidence support this notion: (1) components of the RA signaling pathway are expressed in developing *rugae palatinae* and in the inter-rugal epithelium; (2) *Cyp261a1* expression is enriched in the inter-rugal epithelium; (3) in the inter-rugal epithelium of the *ShhCreER^T2^/Shh^f^* mutant palates *Cyp26a1* expression is severely diminished and *RARg* expression is enhanced; and (4) loss of SHH signaling causes abnormal patterning of *rugae palatinae* [86], this study similar to that engendered by exposure of the developing palate to excess RA [94]. These findings suggest that RA signaling promotes the formation of *rugae palatinae* and that one mechanism by which SHH inhibits *rugae* formation is through the attenuation of RA signaling.

### 2.3. SHH Signaling Is Required for Cyp26 Expression in Other Developing Structures

To explore whether *Cyp26* expression requires SHH inputs in other SHH-dependent developing structures, such as the genital tubercle and embryonic teeth known to express factors involved in RA signaling, we analyzed these organs in SHH-deficient and control embryos.

The genital tubercle (GT), primordium of the penis and clitoris, consists of a mesenchyme covered by ectoderm and a ventral midline structure, the urethral plate epithelium. The urethral plate epithelium derives from the endoderm of the cloaca and generates the entire penile urethra [96]. Previous work established a crucial role for SHH signaling for normal development of the genitourinary system, including proximal-distal outgrowth of the GT and formation of the urethral tube [1,97,98,99,100]. *Shh* expression begins in the cloacal membrane before the onset of GT development, and during GT outgrowth SHH is produced by the urethral plate epithelium and signals to the mesenchyme and ventral ectoderm of the tubercle [96,97,98]. Loss of SHH signaling in the developing GT generates various anomalies, including developmental arrest, hypoplasia due to stunted proximal-distal outgrowth, and/or hypospadias [97,98,99,100].

**Figure 6:**
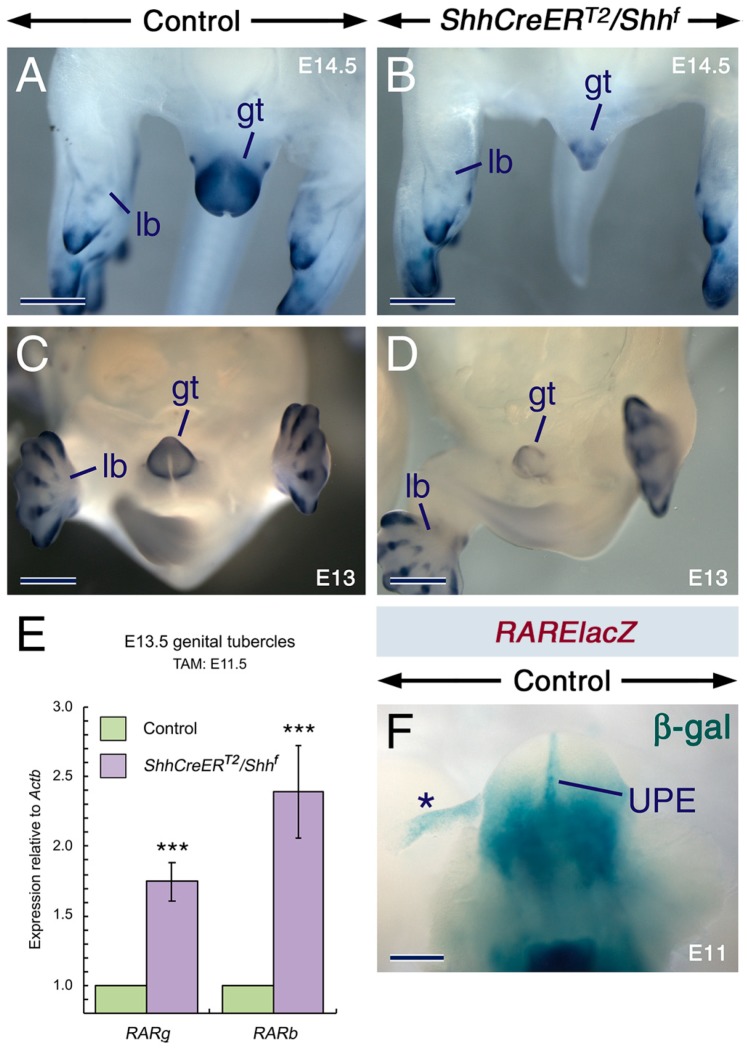
Loss of SHH signaling in the developing genital tubercle causes downregulation of *Cyp26b1* expression and enhancement of retinoic acid signaling. (**A**–**D**) Representative *Cyp26b1* whole-mount in situ hybridization with riboprobes (purple). E14.5 control (**A**; *n* = 2) and *ShhCreER^T2^/Shh^f^* mutant (**B**; *n* = 2) embryos first exposed to tamoxifen (TAM) at E12. E13 control (**C**; *n* = 2) and *ShhCreER^T2^/Shh^f^* mutant (**D**; *n* = 2) embryos first exposed to TAM at E11.5. Diminished *Cyp26b1* hybridization signals in the genital tubercle (gt) of the mutants. Note that *Cyp26b1* signals are not altered in chondrogenic condensations within limb buds (lb) of the mutant as these cellular condensations do not express *Shh*. (**E**) RT-qPCR analysis for *RARb* and *RARg* relative to *Actb* (β-actin) in genital tubercles from E13.5 control and *ShhCreER^T2^/Shh^f^* mutant embryos first exposed to TAM at E11.5. Upregulation of *RARb* (*p* = 0.0009) and *RARg* (*p* = 0.0002) in the mutant (*n* = 8 and *n* = 7 for *RARb* and *RARg*, respectively) as compared to the control (*n* = 8 and *n* = 7 for *RARb* and *RARg*, respectively) genital tubercles. Data are mean values ± standard deviation; ***: *p* < 0.001. (**F**) β-galactosidase (β-gal) histochemistry revealing retinoic acid activity in the genital tubercle of control embryos carrying the *RAREhsplacZ* transgene (*n* = 7). Asterisk in **F** indicates an artefact due to tissue detachment. UPE, urethral plate epithelium. Scale bars: 300 µm (**F**) and 500 µm (**A**–**D**).

Strikingly, rodent embryos exposed to teratogenic doses of vitamin A or RA exhibit GT anomalies [61], mimicking those caused by loss of SHH signaling [97,98,99,100], including GT agenesis and stunted outgrowth of the GT. Components of the RA signaling cascade are expressed before and during outgrowth of the GT. *Aldh1a2* is expressed in the cloacal membrane and urethral plate epithelium [48,49,101], all three *RARs* are expressed in the urethral plate epithelium and in the mesenchyme of the GT [50,51,102,103], and *Cyp26b1* is expressed in the GT mesenchyme [69]. Furthermore, RA activity is readily detectable in the urethral plate epithelium and proximal GT mesenchyme (Figure 6F).

To determine whether *Cyp26b1* expression in the GT requires SHH signaling, we compared the expression of *Cyp26b1* in TAM-treated control and *ShhCreER^T2^/Shh^f^* mutant embryos and found down-regulation of *Cyp26b1* expression in the GT of the mutants (Figure 6A–D). As previously described [97,98,99], the mutants exhibited hypoplastic GT (Figure 6A–D). Furthermore, RA signaling was enhanced in the mutant GT as shown by significant enhancement of *RARb* and *RARg* expression levels (Figure 6E). Thus, like in the limb bud [38], in the developing GT SHH inputs are required for modulating RA activity through maintenance of proper levels of *Cyp26b1* expression.

*Cyp26b1^n/n^* embryos display enhanced RA signaling in the GT and exhibit a range of anomalies of external genitalia, including enlarged width due to increased proliferation of the GT mesenchyme [101]. Yet, unlike mouse embryos deficient in SHH signaling [97,98,99,100] (this study), the *Cyp26b1^n/n^* mutants have intact proximal-distal outgrowth of the GT [101]. The major function of SHH in the GT mesenchyme is to maintain proper rates of mesenchymal cell proliferation required for proximal-distal outgrowth [96]. The lack of abnormal proximal-distal outgrowth of the *Cyp26b1^n/n^* mutant GT is likely due to that *Cyp26b1* ablation occurs in the presence of a functional *Shh* gene, a condition that differs from that of the TAM-induced *ShhCreER^T2^/Shh^f^* mutant GT, in which diminished *Cyp26b1* expression occurs in the absence of SHH inputs. In fact, in *Cyp26b1^n/n^* embryos *Shh* expression in the urethral epithelium and SHH signaling in the GT mesenchyme were found to be upregulated, as a result of increased RA signaling [101]. The phenotype of the GT in the *Cyp26b1^n/n^* mutants is also different from that of rodent embryos exposed to teratogenic doses of vitamin A or RA [61], as in the latter the GT fails to form or is truncated, mimicking the anomalies induced by loss of SHH signaling. A likely explanation for these differences is that in the *Cyp26b1^n/n^* mutants, the GT is exposed to RA emanating from endogenous sources, leading to upregulation of *Shh* expression in this organ [101]. By contrast, in embryos exposed to excess exogenous retinoids the GT is exposed to overwhelming levels of RA. Since teratogenic levels of RA are known to abolish *Shh* expression [32,104], it is possible that in the GT of embryos overexposed to exogenous retinoids, SHH signaling is reduced or lost. Thus, in these embryos, combined loss of SHH signaling and enhanced RA signaling may lead to conditions resembling those that occur upon the genetic loss of SHH signaling in the GT. Taken together, these observations suggest that RA bio-availability must be precisely controlled to ensure normal development of the GT.

Mouse models revealed the importance of SHH signaling during odontogenesis. Loss of SHH signaling in developing teeth of *K14-Cre/Shh^n/f^*, *K14-Cre/Smo^n/f^* and *Evc^n/^*^n^ mutant embryos generates tooth anomalies, including abnormally small and misshapen teeth with enamel defects [41,105,106] and failure of differentiation of enamel-producing ameloblasts, epithelial cells that differentiate from the inner dental epithelium [41,105]. SHH, is produced by the dental epithelium and signals within the dental epithelium and to the dental mesenchyme [41,105]. Developing teeth express genes encoding components of the RA pathway, including RALDHs [48,107], CYP26A1 [69], CYP26C1 [67], and RARs [108]. Remarkably, exposure of mice to excess RA generates enamel defects and abnormal ameloblast differentiation [109], and in vitro exposure of embryonic mouse teeth to supraphysiological levels of RA leads to formation of misshapen teeth [110]. However, physiological levels of RA seem to be required for normal tooth formation, since vitamin A deficiency in rats causes a range of defects, including enamel hypoplasia, abnormal dentine formation, and metaplasia of dental epithelia [111,112,113].

**Figure 7:**
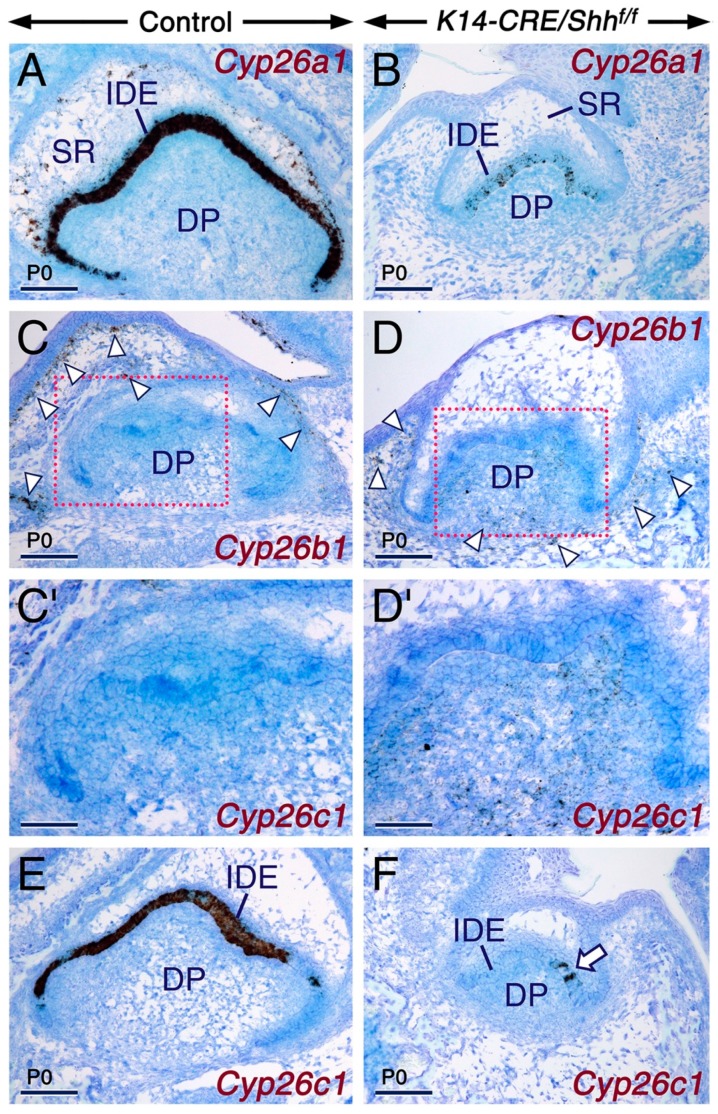
SHH signaling is required for maintenance of the expression of *Cyp26a1* and *Cyp26c1* in the developing tooth. (**A**–**F**). Representative *Cyp26a1* (**A**,**B**; *n* = 2 controls and *n* = 2 mutants), *Cyp26b1* (**C**,**D**; one control and one mutant) and *Cyp26c1* (**E**,**F**; *n* = 2 controls and *n* = 2 mutants) in situ hybridization (black) with oligonucleotide probes in frontal sections across developing first molars from control (**A**,**C**,**E**) and *K14-CRE/Shh^f/f^* mutant (**B**,**D**,**F**) newborn (P0) mice. **C’** and **D’** are magnified views of the boxed areas in **C** and **D**, respectively. The mutant molars show severely diminished *Cyp26a1* hybridization signals in the inner dental epithelium (IDE) and abolished *Cyp26a1* expression in cells of the stellate reticulum (SR). Note also the severely reduced domain of *Cyp26c1* expression in the IDE of the mutant tooth (arrow in **F**). The mutant molars (**D**) exhibit ectopic expression of *Cyp26b1* in the dental papilla mesenchyme (DP). *Cyp26b1* expression in cells of the developing alveolar bone (arrowheads in **C** and **D**) is unaltered in the mutant. Scale bars: 100 µm (**A**–**D**,**E**,**F**) and 50 µm (**C’**,**D’**).

We found that developing teeth from *K14-CRE/Shh^f/f^* mutant mice exhibit severe downregulation of *Cyp26a1* and *Cyp26c1* expression levels in the inner dental epithelium (Figure 7). However, compared to control teeth that showed an absence of *Cyp26b1* expression in the dental papilla mesenchyme (Figure 7C,C’), consistent with previous findings [69], the mutant teeth exhibited ectopic *Cyp26b1* hybridization signals in this tissue (Figure 7D,D’). By contrast, *Cyp26b1* expression in osteoblast progenitors, including in the developing alveolar bone at the periphery of developing teeth [69], was as expected unaltered in the mutants (Figure 7C,D) as these cells do not express Keratin 14 and SHH.

The dependence of *Cyp26b1* expression on SHH signaling seems to be context-dependent. While loss of SHH signaling in the developing limb [38], genital tubercle, and palate (this study) leads to diminished levels of *Cyp26b1* expression, *Cyp26b1* transcript levels are enhanced in tongue mesenchyme of SHH-deficient embryos [40] (see also Figure S4), and *Cyp26b1* is expressed ectopically in the dental papilla mesenchyme of *K14-CRE/Shh^f/f^* mutant molars.

### 2.4. Conclusions

Previous studies in the embryonic limb [38] and tongue [40] together with our present findings in several developing structures showed that SHH signaling abates RA signaling through the maintenance/reinforcement of *Cyp26* expression. Thus, during development of various organs, SHH uses this same strategy to antagonize RA signaling. Furthermore, loss of SHH signaling in the developing tongue [40], tail, secondary palate, genital tubercle, and tooth (this study) causes these structures to develop defects that are remarkably akin to those engendered by genetically or pharmacologically induced overactivation of RA signaling. A literature search revealed strikingly similar congenital anomalies caused by deregulation of Hedgehog and RA signaling (Table S1), suggesting that antagonism between the two pathways may be a common phenomenon.

It is unlikely that SHH directly induces the initial expression of *Cyp26* transcripts, since experiments showed that the Smoothened agonist SAG reinforces *Cyp26a1* expression in *Cyp26a1*-expressing tissues but fails to induce *de novo* expression of this gene in *Cyp26a1*-non-expressing tissues [40] (this study). Which factor(s) whose activities are modulated by SHH signaling directly regulate *Cyp26* expression, and thus RA activity, remain to be identified.

Delineating how cell signaling cascades interact to control tissue patterning, cell fate specification and organogenesis is key to understanding the etiopathogenesis of congenital malformations and malignancies, and knowledge of developmental pathway interactions constitutes a basis for regenerative medicine. Our findings in the developing tail provide a probable, mechanistic explanation for the tail anomalies engendered by loss of SHH signaling, that is, involvement of aberrant enhancement of RA signaling in the genesis of these malformations.

Human embryos develop a tail bud and a transient tail, the latter being normally fated to regress [114,115] through apoptosis [116]. A congenital midline malformation known as “human tail” [114,117,118,119] has been suggested to result from failure of regression of the embryonic tail [115]. Currently, the etiology of “human tail” and underlying molecular mechanisms leading to this anomaly are unknown. Our findings in mouse embryos not only provide insights into the interplay between signaling pathways in the control of development of the caudal region of the embryo, but also provide valuable information for future work aiming at deciphering the etiopathogenesis of “human tail”.

Besides their role in tissue patterning, the Hedgehog and RA pathways play crucial roles during organogenesis and postnatal tissue homeostasis. Compelling evidence suggests that Hedgehog signaling promotes cell proliferation and cell survival [1,19], whereas RA inhibits cell proliferation and induces differentiation and/or apoptosis [3,54,65,78,79,120]. In human multiple myeloma, a B cell malignancy, SHH derived from myeloma plasma cells has been shown to antagonize RA activity through upregulation of CYP26A1 [39]. Altogether these observations prompt the question of whether in other tumor types in which Hedgehog signaling is pathologically upregulated [17,18,121], RA signaling is mitigated through Hedgehog-dependent CYP26-mediated clearance of RA, providing favorable conditions for growth and survival of tumor cells.

## 3. Materials and Methods

### 3.1. Ethics Statement

The procedures involving the use of mice were reviewed and approved by the Animal Research Ethics Committee in Göteborg, Sweden (Dnr. 230-2010 (29 September 2010), 174-2013 (12 November 2013) and 40-2016 (27 April 2016)). Mouse experiments were also carried out under approved protocols in strict accordance with the policies and procedures established by the University of California, San Francisco (UCSF) Institutional Animal Care and Use Committees (UCSF protocol AN084146 re-approved on 05 March 2019).

### 3.2. Mouse Lines

The *K14-CRE/Shh^f/f^* mutant, the *ShhGFPCRE/Smo^f/f^* mutant, the tamoxifen (TAM)-inducible *ShhCreER^T2^/Shh^f^* mutant, and the *Shh^n/n^* mutant embryos as well as their control littermates were generated and identified as described previously [40,41,42,43]. Control and *ShhGFPCRE/Smo^f/f^* mutant embryos carrying the *RAREhsplacZ* transgene [73] were generated as described [40]. For CRE-mediated ablation of *Shh* in *ShhCreER^T2^/Shh^f^* embryos, pregnant females were treated with intraperitoneal injections of TAM every other day (excluding the day of embryo harvest) as described [40].

### 3.3. Histology, Immunohistochemistry, In Situ Hybridization, β-Galactosidase Histochemistry and RT-qPCR

Tissues and organs were processed for histology (Alcian blue van Gieson staining), immunohistochemistry, in situ hybridization, and β-galactosidase histochemistry as described previously [40]. Rabbit antibody targeting cleaved Lamin A (small subunit; 1:1000 dilution) was obtained from Cell Signaling Technology (Danvers, MA, USA). Rabbit monoclonal (MAB) antibody against FOXA1 (1:5000 dilution) was from Abcam (Cambridge, UK). For detection of *Cyp26b1* and *Cyp26c1* transcripts in tissue sections, oligonucleotide probes targeting *Mm-Cyp26b1* (NM_175475.3; target sequence: 460-1308) and *Mm-Cyp26c1* (NM_001105201.1; target sequences: 21-1134) were used. For RT-qPCR assays, the entire tail per embryo, the entire genital tubercle per embryo, and a pair of palatal shelves per embryo were analyzed. *Actb* (β-actin) was used as a reference gene for RT-qPCR data. Primers and conditions for RT-qPCR, other probes used for in situ hybridization, and other antibodies have been described [40].

### 3.4. In Vitro Explant Cultures and Quantification of Apoptosis

*Shh^f/f^* females were mated with *ShhCreER^T2^* males. The pregnant females received an intraperitoneal injection of TAM [40] to induce in utero CRE-mediated *Shh* deactivation at embryonic day 10 (E10). The next day (E11), tails/pelvic girdles were dissected from control and *ShhCreER^T2^/Shh^f^* mutant embryos and cultivated in vitro in an organ culture system as described previously [40]. The medium contained 2.5 and 1.25 µM 4-OH-TAM (4-Hydroxytamoxifen, Sigma-Aldrich, Stockholm, Sweden) during the first and second days of culture, respectively, to enable continuation of CRE-mediated ablation of *Shh* in vitro. The explants were cultivated for a total period of 3 days in the presence of vehicle control (DMSO) or 12.5 µM BMS493, a pan-RAR inverse agonist (Tocris Bioscience, Abingdon, UK). Thereafter, the explants treated with DMSO or BMS493 were processed for Keratin 8 immunohistochemistry. For quantification of apoptosis, sections of tail explants cultivated as described above were processed for immunostaining for cleaved Lamin A to visualize apoptotic cells. Apoptotic epithelial and mesenchymal cells in sections of tail explants from controls and *ShhCreER^T2^/Shh^f^* mutants were counted in the caudal portion of the tail through a ×20 objective. Student’s *t*-test was used for statistical analysis.

For *Cyp26a1* whole-mount in situ hybridization, tails/pelvic girdles were dissected from E11.5 control embryos and cultivated for 24 h in vitro under conditions described previously [40] in the presence of DMSO or 0.2 µM SAG, a small molecule agonist of Smoothened [122].

## Supplementary Materials

Supplementary materials can be found at https://www.mdpi.com/1422-0067/20/9/2275/s1. Figure S1. Loss of SHH signaling in the developing palate of *ShhCreER^T2^/Shh^f^* mutant embryos. Figure S2. The *ShhCreER^T2^/Shh^f^* mutant embryos display cleft palate. Figure S3. RALDH1-3 proteins are produced in the developing palate of control and *ShhCreER^T2^/Shh^f^* mutant embryos. Figure S4. Diminished *Cyp26b1* hybridization signals in the palatal mesenchyme upon loss of SHH signaling. Table S1. Phenotypes caused by loss of Hedgehog signaling and enhancement of retinoic acid signaling in animal models. References [123,124,125,126,127,128,129,130,131,132,133,134,135,136,137,138,139,140,141,142,143,144,145,146,147,148,149,150,151,152,153,154,155,156,157,158,159,160,161,162,163,164,165,166,167,168,169,170,171,172,173,174,175,176,177,178,179,180,181,182,183,184,185,186,187,188,189,190] are cited in the Supplementary Materials files.

## Abbreviations

BMS493	Pan Retinoic acid receptor antagonist
CRE	(Cyclization recombination) DNA recombinase
CYP26A1,B1,C1	Cytochrome P450 isoenzymes A1, B1 and C1
*f*	Floxed allele
FGF	Fibroblast growth factor
FNP	Frontonasal process
FOXA1/*Foxa1*	Forkhead box protein A1 protein/gene
GLI1-3	Glioma-associated oncogene family members 1, 2 and 3
IHH	Indian hedgehog
ISH	In situ Hybridization
K8	Keratin 8
K14	Keratin 14
*LacZ*	Gene encoding *E. Coli* β-galactosidase
*n*	Null allele
SAG	Smoothened agonist
SHH/*Shh*	Sonic Hedgehog protein/gene
SMO/*Smo*	Smoothened protein/gene
PS	Palatal shelf/shelves
Ptch1	Patched 1
RA	Retinoic acid
RALDH/*Aldh1a*	Retinaldehyde dehydrogenase protein/gene
RAR	Retinoic acid receptor
RARE	Retinoic acid response element
RT-qPCR	Reverse transcription quantitative polymerase chain reaction
RXR	Retinoid X receptor
TAM	Tamoxifen
WMISH	Whole-mount in situ hybridizationWingless/integrated 3a
4-OH-TAM	4-hydroxytamoxifen
