# Supplementary material for: Sonic Hedgehog Signaling Is Required for Cyp26 Expression during Embryonic Development
Source: Int J Mol Sci. 2019 May 8;20(9):2275. doi: 10.3390/ijms20092275 (PMC6540044; doi:10.3390/ijms20092275)
Supplement: Supplementary file 1 [file ijms-20-02275-s001.zip › Suppl Rev 2/Supplemental Figs.pdf]

Supplementary Materials

Supplemental Figure Legends

<u>Figure S1</u>. Loss of SHH signaling in the developing palate of *ShhCreER*^{T2}*/Shh*^{f} mutant embryos

(**A-F**) Representative *in situ* hybridization with oligonucleotide probes targeting *Gli1* (A-D; dark brown) and *Ptch1* (E,F; brown), two well-established direct targets of Hedgehog signaling [1], in parasagittal sections of palates (anterior palatal region towards the left of the panels) from E13.5 control (A,C,E; n=3 for each gene) and *ShhCreER*^{T2}*/Shh*^{f} mutant (B,D,F; n=3 for each gene) embryos first exposed to tamoxifen at E10-5-E11. In the mutant palates deactivation of the *Shh* gene in the palatal epithelium caused loss of SHH signaling in the palatal epithelium and palatal mesenchyme, as indicated by severely diminished *Gli1* and *Ptch1* hybridization signals in these tissues. The arrows in C,D,E and F indicate *rugae palatinae*. md, mandible, PS, palatal shelf. Scale bars: 200 µm (A,B) and 100 µm (C-F).

<u>Figure S2.</u> The *ShhCreER*^{T2}*/Shh*^{f} mutant embryos display cleft palate

(**A-F**) Representative Alcian blue van Gieson staining of frontal sections across the anterior (A,D), middle (B,E), and posterior (C,F) palatal regions of E16 control (A-C; n=3) and *ShhCreER*^{T2}*/Shh*^{f} mutant (D-F; n=3) embryos first exposed to tamoxifen at E10.5. The mutant displays cleft palate. P, palate; PS, palatal shelf; T, tongue. Scale bars: 500 µm.

<u>Figure S3</u> RALDH1-3 proteins are produced in the developing palate of control and *ShhCreER*^{T2}*/Shh*^{f} mutant embryos

(**A-R'**) Representative immunostaining (dark purple) for RALDH1 (A-F'), RALDH2 (G-L') and RALDH3 (M-R') in frontal sections across the anterior, middle and posterior regions of the developing palate at E13.5 from a control embryo (A-C',G-I',M-O') and a *ShhCreER*^{T2}*/Shh*^{f} mutant embryo (D-F',J-L',P-R') embryos first exposed to tamoxifen at E10.5-E11. A'-R' are magnified views of the boxed areas in A-R. The palatal periderm (arrows in A',B',C',D',E' and F'), a subset of cells in the

mesenchyme underlying *rugae palatinae* (arrowheads in B') and the epithelium of the bend region of the palate (arrows in A,B,D and E) are RALDH1-positive. The palatal periderm (arrows in G' and K'), *rugae palatinae* (H'), mesenchymal cells within the palatal shelves (G'-K'), the bend region of palatal shelves (arrows in G,H,J and K), and cells surrounding blood vessels (arrowheads in G',H', and J') are RALDH2-positive. Cells surrounding blood vessels (arrowheads in M',N',P',Q' and R') as well as the epithelium and mesenchyme of the bend region of palatal shelves (arrows in M,N,P and Q) are RALDH3-positive. PS, palatal shelf; R, *rugae palatinae*; T, tongue. Scale bars: 200 μm (A-R) and 50 μm (A'-R').

Figure S4. Diminished *Cyp26b1* hybridization signals in the palatal mesenchyme upon loss of SHH signaling

(**A-F**). Representative *Cyp26b1 in situ* hybridization (dark brown) with oligonucleotide probes in frontal sections across the anterior, middle, and posterior regions of palates from E13.5 control (A-C; n=2) and *ShhCreER*^{T2}*/Shh*^{f} mutant (D-F; n=2) embryos first exposed to tamoxifen at E10.5-E11 (see also Figure 4). A'-F' are magnified images of the boxed areas in A-F. In the mutants *Cyp26b1* hybridization signals are diminished in the palatal shelf mesenchyme. In the *ShhCreER*^{T2}*/Shh*^{f} mutant embryos SHH signaling is abrogated in the epithelium and mesenchyme of the tongue [40]. The mesenchyme of the mutant tongue shows abnormally enhanced *Cyp26b1* hybridization signals as compared to the mesenchyme of the control tongue (arrows). T, tongue. Scale bars: 200 μm (A-F) and 100 μm (A'-F').

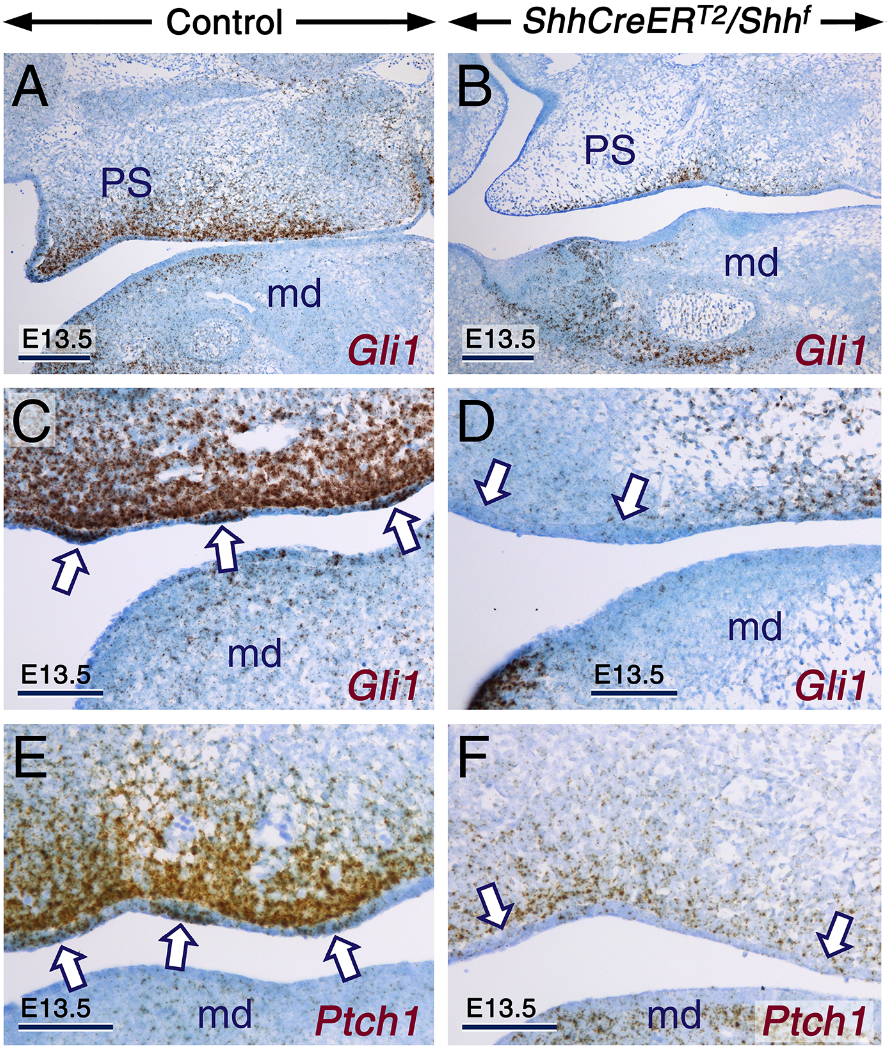

Control
ShhCreER^{T2}/Shh^{f}
A
PS
md
E13.5
Gli1
B
PS
md
E13.5
Gli1
C
md
E13.5
Gli1
D
md
E13.5
Gli1
E
E13.5
md
Ptch1
F
E13.5
md
Ptch1

Figure S1

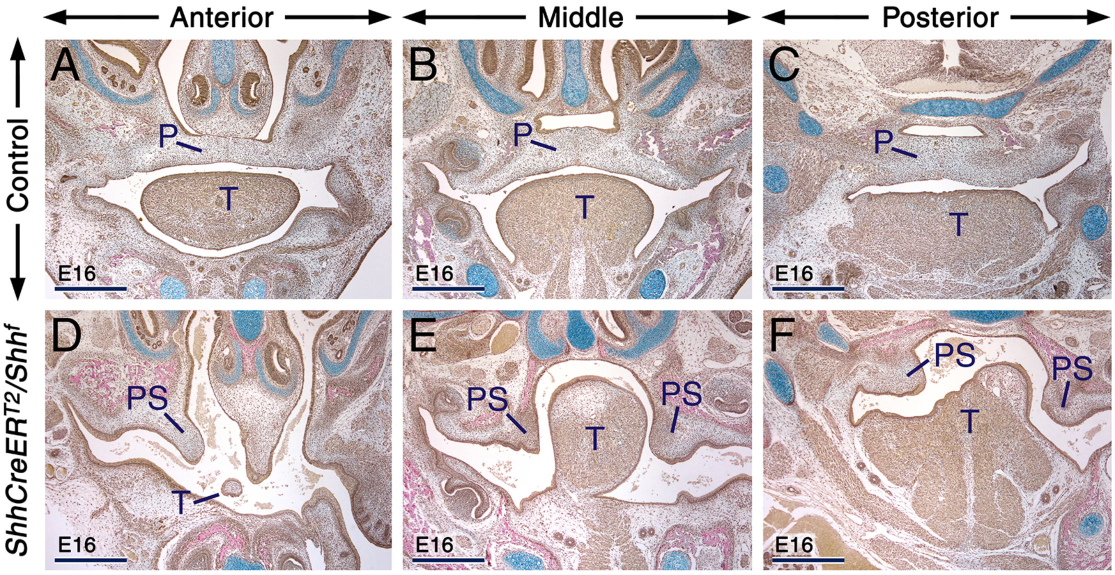
Anterior
Middle
Posterior
Control
ShhCreER^{T2}/Shh^{f}
A
B
C
D
E
F
P
T
PS
E16

Figure S2

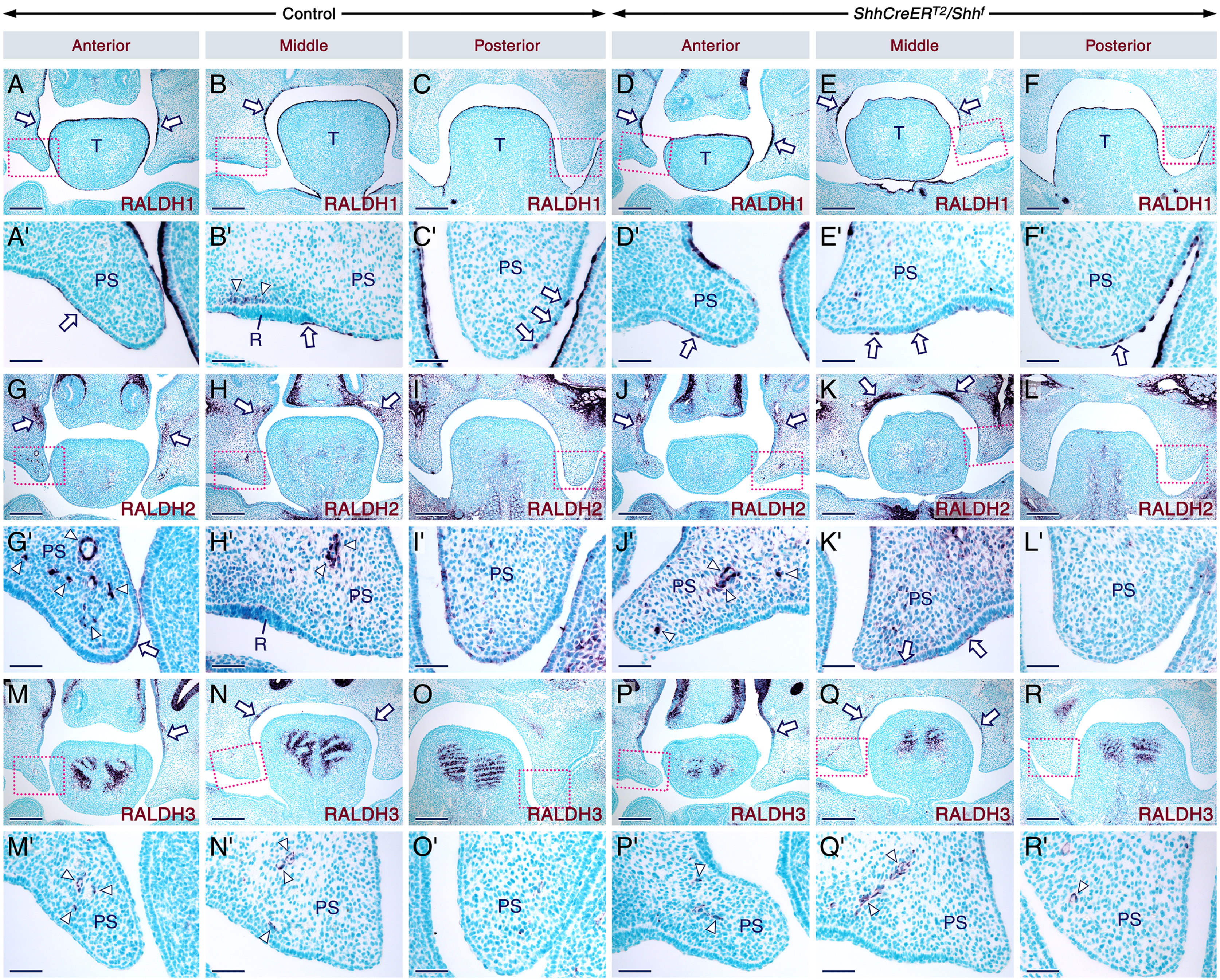
Control
ShhCreERT2/Shhf
Anterior
Middle
Posterior
Anterior
Middle
Posterior
RALDH1
RALDH2
RALDH3
T
PS
R

Figure S3

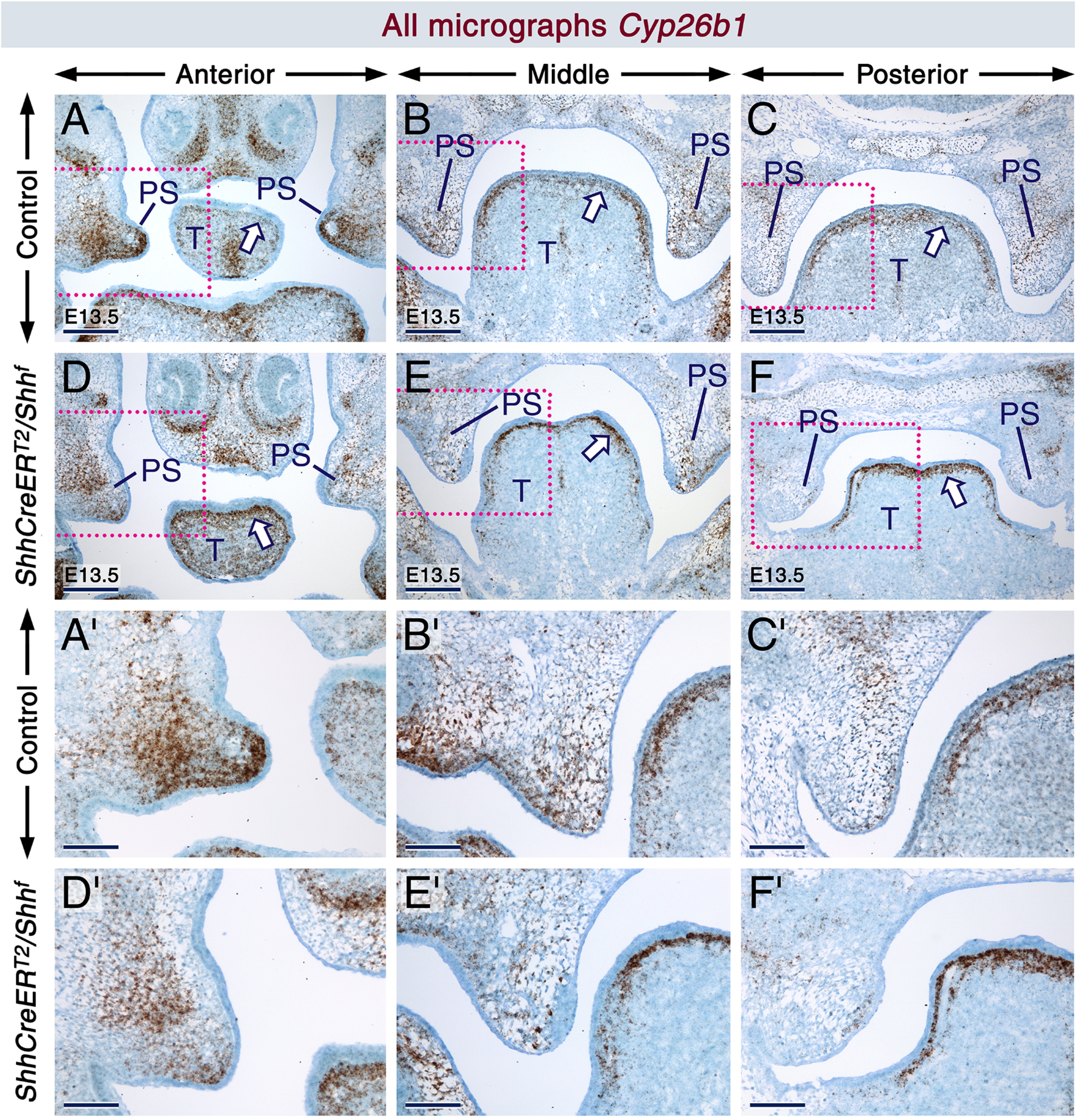
All micrographs Cyp26b1
Anterior
Middle
Posterior
Control
ShhCreERT2/Shhf
A
B
C
D
E
F
A'
B'
C'
D'
E'
F'
PS
T
E13.5

Figure S4
