# Supplementary material for: Sonic Hedgehog Signaling Is Required for Cyp26 Expression during Embryonic Development
Source: Int J Mol Sci. 2019 May 8;20(9):2275. doi: 10.3390/ijms20092275 (PMC6540044; doi:10.3390/ijms20092275)
Supplement: Supplementary file 1 [file ijms-20-02275-s001.zip › Suppl Rev 2/Table S1 .pdf]

Table S1. Phenotypes caused by loss of Hedgehog signaling and enhancement of retinoic acid signaling in animal models

Pathways	Main gene expression sites in mouse embryos [references]	Phenotype of animal models [references]
	**Early embryogenesis**	
Hedgehog signaling	*Shh*: node, midline mesenchyme of the head process, notochord, and gut endoderm, and *Ihh*: posterior part of the node, visceral endoderm, gut endoderm, and CMC [1,71,72,123-125]. *Ptch1* and *Gli1*: Hh-responding tissues [1]. *Cdo* and *Boc*: Hh-responding tissues [126] . *Gas1*: Hh-responding tissues [127,128].	- $Smo^{n/n}$ and $Shh^{n/n}/Ihh^{n/n}$ mice: viable up to E9.5, failure of establishment of LR asymmetry, abnormal heart looping and sclerotomal development, ventral cyclopia, and HPE [1,125].<br>- $Shh^{n/n}$ mice: cyclopia, HPE, midbrain truncation, agenesis of all vertebrae, degeneration of the notochord, caudal truncation (short and kinky tail devoid of tail vertebrae), hypoplasia of BA1, hypoplasia (micrognathia) and medial clefting of the mandible, severe reduction of head size and of the frontonasal region [44], impaired heart looping, and several laterality defects, including pulmonary left isomerism (bilaterally monolobed lungs) [31].<br>- $Gas1^{n/n}/Boc^{n/n}$ mice: HPE, cleft lip, cleft palate, and clefting of the pharyngeal tongue [127].<br>- $Cdo^{n/n}$, $Cdo^{n/n}$/$Boc^{n/n}$, $Cdo^{n/n}/Boc^{+/n}$ and $Gas1^{n/n}$ mice: microform HPE[IP] [126,127,129,130].<br>- $ShhGFPCRE/Smo^{f/f}$ and TAM-induced $ShhCreER^{T2}/Shh^{f}$ mice: degeneration of the notochord and caudal truncation (short and kinky tail, agenesis of vertebrae caudal to lumbar vertebrae, including tail vertebrae) [42; this study].
RA signaling	*Aldh1a1*: ventral mesencephalon, dorsal retina and OV [131]. *Aldh1a2*: presomitic and somitic mesoderm, LPM, and cloaca [22,48,49,132,133]. *Aldh1a3*: head ectoderm, olfactory placodes/pits, optic vesicle, ventral retina, RPE, OV, and Rathke's pouch [22,131,134]. *Rdh10*: LPM and somites [135]. *Cyp26a1*: epiblast, M of head process, embryonic mesoderm, anterior neural plate (transient), visceral and parietal endoderm, r2, cervical M, NCC progenitors of cranial nerve ganglia, tail bud structures (neural plate posterior to the caudal neuropore, endoderm and mesoderm), caudal sclerotomes, OV, and E and M of the FNP, [25,27,54,56,57,67]. *Cyp26b1*: hindbrain (r2-r6), M of limb buds [57,68], spinal cord and CMC [69]. *Cyp26c1*: M of head process, r2 and r4, OV, and BA1 [67]. *RARa, RXRa* and *RXRb*: ubiquitous, *RARb, RARg* and *RXRg*: tissue-specific, and *RARg*: primitive streak, FNP, BA1, trunk and caudal neural plate, presomitic mesoderm, tailbud mesoderm, and CMC [22,78].	- $Cyp26a1^{n/n}$ mice: caudal truncation (absent[IP] or short and kinky tail, malformation or agenesis of lumbo-sacral skeletal elements, and agenesis of tail vertebrae), imperforate anus, exencephaly[IP] (NTD between the forebrain and hindbrain), hindbrain mispatterning, abnormal heart looping, hypoplasia of BA1, overgrowth of neural tissue, and fusion of trigeminal and facial nerve ganglia [25,27,59].<br>- $Cyp26a1^{n/n}$ mice exposed to sub-teratogenic doses of RA: severe head truncation and abnormal vasculogenesis [136].<br>- $Cyp26a1^{n/n}/Cyp26c1^{n/n}$ mice: early embryonic lethality (before E11), partial or total duplication of body axis[IP], forebrain truncation, hindbrain mispatterning, severe reduction of head size and of the frontonasal region, hypoplasia of BA1, exencephaly (NTD between the forebrain and hindbrain), agenesis of trigeminal ganglia, and hypoplastic cranial ganglia VII and VIII [3,27,137].<br>- $Cyp26a1^{n/n}/Cyp26b1^{n/n}/Cyp26c1^{n/n}$ mice: early embryonic lethality (before E10) partial or total duplication of body axis[IP] [137].
Pharmacological enhancement of RA signaling	NA	- Overexposure to exogenous RA or vitA (rodents: mice, rats and/or hamsters): cyclopia and/or severe HPE [62,138].<br>- Overexposure to exogenous RA or vitA (rodent and chick embryos): HPE, cyclopia [32] and microform HPE [61-65,104,138].<br>- Overexposure to exogenous RA and retinoids (rodents): caudal truncation (absent tail, short and kinky tail, absent tail vertebrae) [61-65,139,140], degeneration of the notochord [66], and exencephaly [61-63,141,142].<br>- Overexposure to exogenous RA at E4.5-E5.5 (mice): limb and lower body duplications [143].<br>- Overexposure to exogenous RA (hamsters): laterality defects, including pulmonary left isomerism (bilaterally monolobed lungs) and visceral *situs inversus*, caudal truncation (agenesis or truncation of the tail), HPE, cyclopia, and microform HPE (cebocephaly) [61].<br>- Overexposure to etretinate (mice): caudal truncation (short tail) [144,145].

Craniofacial structures		
Hedgehog signaling	*Shh*: various craniofacial epithelia and nasal placodes/pits [1,19,89]. *Ihh*: CMC, caniofacial skeleton, cranial sutures [1,19,71,72]. *Ptch1* and *Gli1*: Hh-responding tissues [1,19,71,72,87,89]. *Cdo, Boc* and *Gas1*: Hh-responding tissues [126,127].	- *Shh*$^{n/n}$ mice: cyclopia, HPE, hypoplasia of BA1, severe reduction of head size and of the frontonasal region, aglossia (tongue agenesis), near absence of the cranial skeleton, and micrognathia and medial clefting of the mandible [44].<br>- *Gli2*^{zfd}/*Gli2*^{zfd} (loss of *Gli2* function) mice: cleft palate and agenesis of maxillary and/or lower incisors [146].<br>- *Cdo*$^{n/n}$ mice: microform HPE$^{(IP)}$ (SMMCI, cebocephaly and mid-facial hypoplasia) [126].<br>- *Cdo*$^{n/n}$/*Boc*$^{n/n}$ and *Cdo*$^{n/n}$/*Boc*$^{+/n}$ mice: microform HPE$^{(IP)}$ (cebocephaly and agenesis or abnormal primary palate), mispatterned or cleft palate, and craniofacial skeletal defects [126,130].<br>- *Gas1*$^{n/n}$ mice: microform HPE (SMMCI) and cleft palate [127,129].<br>- *Gas1*$^{n/n}$/*Boc*$^{n/n}$ mice: lobar HPE (failure of separation of the basal part of frontal lobes), cleft palate, cleft pharyngeal tongue and cleft lip [127].<br>- *Osr2-IresCre/Smo*$^{f/f}$ mice: cleft palate [147].<br>- *Wnt1-Cre/Smo*$^{n/f}$ or *Wnt1-Cre/Smo*$^{f/f}$ mice: agenesis of lower incisors, microform HPE (SMMCI), agenesis or hypoplasia of NCC-derived craniofacial skeletal elements, truncation of Meckel's cartilage, facial skin tags, open eyelids at birth [87], and microglossia and bifid tongue [40,87].<br>- *K14-Cre/Shh*$^{f/f}$ or *K14-Cre/Shh*$^{n/f}$ mice: cleft palate [88,147], choanal atresia, and open eyelids at birth [41].<br>- TAM-induced *ShhCreER*^{T2}/*Shh*^{f} mice: microglossia, bifid tongue (cleft oral tongue), adhesion^{IP} of the LE to the oral E [40], and cleft palate [this study].<br>- *ShhGFPCRE/Smo*$^{f/f}$ mice: adhesion^{IP} of the LE to the oral E [40].<br>- Pharmacological inhibition of Hedgehog signaling in mice: HPE and microform HPE (cebocephaly), cleft lip and cleft palate [148,149].<br>- *Ihh*$^{n/n}$ mice: shortening of the mandible (dentary bone), reduced size of calvarial bones, reduced size but normal ossification of the cranial base [71].
RA signaling	*Aldh1a1-a3*/RALDH1-3: E and/or M of craniofacial structures[40,107,134,150,151, this study]. *Cyp26a1* and *Cyp26c1*: various craniofacial epithelia [40,57,67,69, this study]. *Cyp26a1*: Meckel's cartilage, and E and M of the FNP [56, this study]. *Cyp26b1*: NCC-derived M of various craniofacial structures [57,69, this study]. *RARs* and *RXRs*: ubiquitous [78].	- *Cyp26a1*$^{n/n}$ mice: exencephaly^{IP} (NTD between the forebrain and hindbrain), hindbrain mispatterning, hypoplasia of BA1, and fusion of trigeminal and facial nerve ganglia [25,27,59].<br>- *Cyp26a1*$^{n/n}$ mice exposed to sub-teratogenic doses of RA: severe reduction of head size and abnormal vasculogenesis [136].<br>- *Cyp26a1*$^{n/n}$/*Cyp26c1*$^{n/n}$ mice: early embryonic lethality (before E11), forebrain truncation, hindbrain mispatterning, severe reduction of head size and of the frontonasal region, hypoplasia of BA1, exencephaly (NTD between the forebrain and hindbrain), agenesis of trigeminal ganglia, and hypoplastic cranial ganglia VII and VIII [3,27,137].<br>- *Cyp26b1*$^{n/n}$ mice: cleft palate [68,77], agenesis of lower incisors, close-set maxillary incisors, open eyelids at birth, severe craniofacial skeletal anomalies of NCC-derived structures and of some mesodermally-derived bones, including truncation of Meckel's cartilage, shortening of the mandible, and reduced ossification of calvarial bones [68].<br>- *Cyp26b1* hypomorphic zebrafish: craniosysnostosis and skeletal defects phenocopying the anomalies in humans with *CYP26B1* mutations [152].
Pharmacological manipulation of RA signaling		- Overexposure to vitA (mice): HPE, cyclopia, microform HPE (SMMCI), agenesis or truncation of Meckel's cartilage, facial skin tags, severe craniofacial skeletal anomalies, cleft palate, micrognathia and medial clefting of the mandible, adhesion of the LE to the oral E, exencephaly [62], absence of eyelids [142], open eyelids in near term fetuses [63], close-set maxillary incisors, and exencephaly [141].<br>- Overexposure to exogenous RA or retinoids (mice and/or rats): cleft palate [153], cleft palate and other craniofacial skeletal anomalies, including micrognathia and medial clefting of the mandible [62,64,93,139,142,154], truncation of Meckel's cartilage [62,63,93,154], aglossia, microglossia [93], microglossia and bifid tongue [154], medially cleft mandible [64], open eyelids in near term fetuses [65], and adhesion of the LE to the oral E [62,142].<br>- Overexposure to exogenous RA (hamsters): microglossia and bifid tongue, micrognathia and medial clefting of the mandible, adhesion of the LE to the oral E, craniofacial skeletal defects, exencephaly, cleft lip and cleft palate, cleft palate, HPE, cyclopia, microform HPE (cebocephaly) and missing eyelids [61].<br>- Overexposure to exogenous RA (chick): microform HPE [104], HPE and cyclopia [32].<br>- Overexposure to etretinate (mice): cleft palate [145].

**Foregut derivatives (oesophagus, trachea and lungs)**		
Hedgehog signaling	*Shh*: E, and *Ptch1* and *Gli1-3*: E and M [1,19].	- *Shh*$^{n/n}$ mice: oesophageal atresia, tracheo-oesophageal fistula, abnormal tracheal cartilage, hypoplastic lungs [31,155,156], and several laterality defects, including pulmonary left isomerism (bilaterally monolobed lungs) [31].<br>- *Gli2*$^{n/n}$ mice: hypoplasia and abnormal lobulation of the lungs [157].<br>- *Gli2*$^{n/n}$/*Gli3*$^{+/n}$ mice: oesophageal atresia, tracheo-oesophageal fistula, severe lung lobulation defects, and abnormal tracheal cartilage [157].<br>- *Gli2*$^{n/n}$/*Gli3*$^{n/n}$ mice: total agenesis of lungs, oesophagus and trachea [157].
RA signaling	*Aldh1a1*: E and M of oesophagus, E of bronchi, tracheal E, and pulmonary parenchyma [107]. *Aldh1a2*: M of oesophagus[48,107] and M of tracheal and lung primordia [158]. *Aldh1a3*: bronchi/pulmonary parenchyma [107]. *Cyp26a1*: foregut endoderm [55], outer M of oesophagus [56], lung buds, main bronchi, tracheal E, and prospective tracheal rings [158]. *Cyp26b1:* lung parenchyma at E16.5 [69]. *RARb* and *RARg*: E and/or M of foregut derivatives [50,78,158,159].	- *Cyp26a1*$^{n/n}$ mice: no defects in foregut derivatives have been reported in these mutants [25,59].<br>- *Cyp26a1*$^{n/n}$/*Cyp26c1*$^{n/n}$ mice: early embryonic lethality (before E11) before development of foregut derivatives [27,137].<br>- *Cyp26a1*$^{n/n}$/*Cyp26b1*$^{n/n}$/*Cyp26c1*$^{n/n}$ mice: early embryonic lethality [137].
Pharmacological enhancement of RA signaling	NA	- Overexposure to exogenous RA or vitA (hamsters): pulmonary left isomerism (bilaterally monolobed lungs) [24,61].<br>- *In* vitro exposure of embryonic lungs to RA (mice): hypoplasic lungs (reduced number of terminal branches) [158].
**Gastrointestinal tract**		
Hedgehog signaling	*Shh*: cloacal endoderm [96], gut endoderm, and epithelia of forestomach, small intestine and colon [19,160]. *Ihh*: gut endoderm, E of GIT from hindstomach to anus [19,160]. *Ptch1* and *Gli1*: mainly in M of GIT [19,160].	- *Shh*$^{n/n}$ mice: intestinal transformation of the stomach, gut malrotation without gut *situs* reversion, overgrown gut villi, annular pancreas[IP], duodenal stenosis[IP], development of ectopic enteric neurons in the small intestine [1,160], imperforate anus (colon terminates in a blind ampulla), and persistent and undivided cloaca [1,160,161].<br>- TAM-induced *ShhCreER*^{T2}/*Shh*^{f} mice: persistent and undivided cloaca [97-100].<br>- *Ihh*$^{n/n}$ mice: gut malrotation without gut *situs* reversion, dilation of small intestine and reduced villus size, annular pancreas[IP], and dilated, thin-walled, aganglionic colon (Hirchsprung's-like phenotype), and aganglionic portions of small intestine[IP][1,160].<br>- *Gli2*$^{n/n}$ or *Gli3*$^{n/n}$ mice: imperforate anus and anal stenosis/atresia [161].<br>- *Gli2*$^{+/n}$/*Gli3*$^{n/n}$ or *Gli2*$^{n/n}$/*Gli3*$^{+/n}$ mice: persistent and undivided cloaca [161].
RA signaling	*Aldh1a1*: E and M of oesophagus, E and outer M of the embryonic GIT [107]. *Aldh1a2*: cloaca [49], outer M of gut [48,107], and M of oesophagus [48,107]. *Aldh1a3*: lamina propria [107]. *Cyp26a1*: cloaca, hindgut, and outer M of oesophagus and stomach [56,69]. *RARa*: ubiquitous, *RARb* and *RARg*: dynamic expression patterns in E and/or M of the developing GIT [50].	- *Cyp26a1*$^{n/n}$ mice: imperforate anus (colon terminates in a blind ampulla) and agenesis of the presumptive rectum [25].<br>- *Cyp26b1*$^{n/n}$ mice: no anomalies of the gastro-intestinal tract have been reported [26,68].<br>- *Cyp26a1*$^{n/n}$/*Cyp26c1*$^{n/n}$ mice: early embryonic lethality (before E11) before development of the GIT [27,137].<br>- *Cyp26a1*$^{n/n}$/*Cyp26b1*$^{n/n}$/*Cyp26c1*$^{n/n}$ mice: early embryonic lethality [137].
Pharmacological enhancement of RA signaling	NA	- Overexposure to exogenous RA (mice): imperforate anus, persistent and undivided cloaca [65], and anal stenosis/atresia [63].<br>- Overexposure to exogenous RA (hamsters): imperforate anus and visceral *situs inversus* [61].<br>- Overexposure to vitA (mice): anal stenosis/atresia [62].<br>- Overexposure to etretinate (mice): imperforate anus [144].

	**Urinary system**	
Hedgehog signaling	*Shh*: urothelium and presumptive ureter (E11.5), distal collecting ducts and ureter (E14.5), inner collecting medullary ducts, E of the renal pelvis and E of the ureter (P0), *Ihh*: nephrogenic E, and *Ptch1*: M adjacent to Hh-expressing epithelia [1,162].	- *HoxB7-Cre/Shh*$^{n/f}$ mice: kidney hypoplasia (deficient kidney stroma due to decreased proliferation and differentiation of smooth muscle cells), hydronephrosis, and hydroureter [162].<br>- *Gli2*$^{n/n}$/*Gli3*$^{+/n}$ mice: horseshoe kidney (fused kidneys) [1].
RA signaling	*Aldh1a2:* mesonephros [49], nephrogenic E [103], kidney M at E11, and after E11 expression in the cortical stroma, comma-shaped bodies, differentiating glomerulus, and Henle's loop [163]. *Aldh1a1:* mesonephros, ureteric bud branches, collecting ducts and outer medulla, and *Aldh1a3:* renal papilla and developing and mature collecting ducts [107,131]. *Cyp26a1:* nephrogenic E and mesonephric ducts [56]. *Cyp26b1:* nephrogenic zone [69]. *RARa*: ubiquitous, and *RARb*: renal stroma and M of the urogenital sinus, ureters and genital tract [78].	- *Cyp26a1*$^{n/n}$ mice: kidney hypoplasia (kidney consisting of a few metanephric tubules) and horseshoe kidney [25,59].
Pharmacological enhancement of RA signaling	NA	- Overexposure to vitA (mice and/or rats): hydronephrosis, agenesis of kidney and ureter, hydroureter and horseshoe kidney [62,142].<br>- Overexposure to exogenous RA (mice and/or rats): kidney hypoplasia, hydronephrosis, hydroureter and horseshoe kidney [63,65,139].<br>- Overexposure to etretinate (mice): hydronephrosis [145].<br>- Overexposure to exogenous RA (hamsters): hypoplastic kidneys, hydronephrosis and hydroureter [61].
	**Male gonad**	
Hedgehog signaling	*Dhh*: Sertoli cell precursors and E of seminiferous tubules, and *Ptch1*: pre-Leydig cells, peritubular cells, germ cells [1,19,164], and epididymal M [164]. *Shh*: epididymal E [164].	- *Dhh*$^{n/n}$ mice: various defects influenced by the genetic background, including development of hypoplastic testes resulting from abnormal formation of seminiferous tubules concomitant with loss of peritubular myoid cells and failure of Leydig cell differentiation, loss of germ cells (in all seminiferous tubules), male infertility, and feminized males^{IP} [1,164].
RA signaling	*Aldh1a1*: Sertoli cells, *Aldh1a2*: mesonephroi, Sertoli cells (low levels of expression after birth), germ cells, spermatocytes and round spermatids, *Aldh1a3*: adult Leydig cells, *Cyp26b1*: Sertoli cells, *Cyp26a1/b1/c1*: peritubular myoepithelial cells, and *RARa* and *RXRb*: Sertoli cells [5,54,165,166].	- *Cyp26b1*$^{n/n}$ mice: hypolastic testes, virtual absence of germ cells, normal Sertoli and Leydic cell development [54], ovo-testes, impaired Leydig cell differentiation, and feminized males [167].<br>- *Amh-Cre/Cyp26b1*$^{sc/sc}$ mice (Sertoli cell-specific ablation of *Cyp26b1* at E15): absence of germ cells in many seminiferous tubules [54,168].
Pharmacological enhancement of RA signaling	NA	- Overexposure to vitA (rats): degeneration of testes [169].
	**External genitalia/Genital Tubercle/Cloaca**	
Hedgehog signaling	*Shh*: cloacal membrane endoderm, urethral plate E, and developing urethral tube [96]. SHH-responding cells: M and ventral ectoderm of the GT [96].	- *Shh*$^{n/n}$ mice: agenesis of external genitalia and persistent and undivided cloaca [96,161].<br>- TAM-induced *ShhCreER*^{T2}/*Shh*^{f} mice: hypoplastic GT (defects in proximal-distal outgrowth) [96-100, this study], hypospadias, persistent cloaca [96-100], hypospadias [97], and agenesis of preputial glands [100].<br>- *Msx2Cre/Smo*$^{f/f}$ mice: Hypospadias [97].<br>-*Msx2-rTA/tetO-Cre/Smo*$^{f/f}$ mice: hypoplastic GT and proximal hypospadias [98].<br>- *Gli2*$^{+/n}$/*Gli3*$^{n/n}$ or *Gli2*$^{n/n}$/*Gli3*$^{+/n}$ mice: persistent and undivided cloaca [161].<br>- *Dermo1-Cre/Smo*$^{f/f}$ mice: hypoplastic GT [98].

RA signaling	*Aldh1a2*: cloaca [49], caudal ventral region adjacent to the GT and around the cloaca (before E12.5), and urethral E (from E12.5 onwards) [48,101]. *Cyp26a1*: weak expression in the urethral plate E, and *Cyp26b1*: M of the GT [69]. *RARa, RARb* and *RARg*: dynamic expression in the M of GT and urethral E [50,51,101,102].	- *Cyp26b1*$^{n/n}$ mice: enhanced SHH and BMP signaling, enlarged and abnormally branched distal urethra, hyperplasia of the preputial E, hyperplasia of the M of the GT, lack of prospective corpus cavernosum, misshapen glans, agenesis of preputial glands, but no defects in proximal-distal outgrowth of the GT [101].
Pharmacological enhancement of RA signaling	NA	- Overexposure to exogenous RA (hamsters): agenesis of external genitalia or hypoplastic GT [61].<br>- Overexposure to exogenous RA (mice): persistent and undivided cloaca [65].
**Cartilage and bone (axial and appendicular skeleton)**		
Hedgehog signaling	-*Ihh*: CMC, prehypertrophic and early hypertrophic chondrocytes [1,71,72]. *Ptch1* and *Gli1*: IHH-responding cells [1,71,170].	- *Ihh*$^{n/n}$ and *Col2-Cre/Smo*$^{n/f}$ mice: severely reduced size of long bones (reduced chondrocyte proliferation, reduced/delayed chondrocyte differentiation, and absence of bone collar) [1,170,171].<br>- Perichondrium-specific ablation of *Smo* in mice: failure of development of a bone collar and primary spongiosa, formation of ectopic cartilage [170].
RA signaling	*Aldh1a2:* perichondrium [48]. RALDH3: prehypertrophic and hypertrophic chondrocytes, RALDH2: proliferating chondrocytes, subsets of bone marrow cells (strong immunostaining), hypertrophic chondrocytes, and osteoblasts (weak immunostaining) [our unpublished data]. *Cyp26a1*: Meckel's cartilage, CMC, and transient expression in the perichondrium [56,69]. *Cyp26b1*: CMC, perichondrium and tendons [69], cranial sutures [152], and epiphysis [172]. *RARa*: ubiquitous, and *RARg*: CMC, perichondrium and chondrocytes [50,78].	- *Cyp26b1*$^{n/n}$ mice: reduced size of long bones (delayed maturation of chondrocytes, failure of development of perichondrium, and absence of *Ihh* transcripts) and radiohumeral synostosis [26,68].<br>- Chondrocyte-specific ablation of *Cyp26b1* in mice: reduced skeletal growth (reduced height of the proliferative zone in the growth plate), VAD diet partially rescued the phenotype [172].
Pharmacological enhancement of RA signaling	NA	- Overexposure to vitA (mice, rats and/or rabbits): agenesis or severe shortening (reduced growth) and deformities of long bones and axial skeletal elements [62,142].<br>- Overexposure to exogenous RA (mice): phenocopies the limb skeletal defects of *Cy26b1*$^{n/n}$ mice [26], and absence of femur and fibula [65].<br>- Overexposure to exogenous RA (hamsters): shortening (reduced growth) and deformities of long bones [61].<br>- Exposure to RARγ agonists (mice): inhibition of chondrogenesis *in vitro* and inhibition of heterotopic ossification in mouse models of *Fibrodysplasia Ossificans Progressiva* [173].
**Epidermis, hair and feather follicles, lingual epithelium and mesenchyme, glands and *rugae palatinae***		
Hedgehog signaling	*Shh*: E of HFs and feather follicles, *rugae palatinae*, and LE [1,19,40,90]. *Ptch1* and *Gli1*: E and M (SHH-responding tissues) [1,19,40,174].	- *Shh*$^{n/n}$, *K14-Cre/Shh*$^{n/f}$ and *K14-Cre/Shh*$^{f/f}$ mice: abnormal growth, morphogenesis and differentiation of HFs, and epidermal hyperplasia [1,174].<br>- *K14-Cre/Smo*$^{f/f}$ mice: epidermal hyperplasia, *de novo* formation of HFs, glandular metaplasia (transformation of interfollicular epidermis and HFs of the ventral skin into mammary glands), and abnormal morphogenesis and differentiation of HFs [174].<br>- *ShhGFPCRE/Smo*$^{f/f}$ mice: glandular metaplasia [cell fate change of the lingual epithelium into minor SGs [40].<br>- *K14-Cre/Shh*$^{f/f}$ and TAM-induced *ShhCreER*^{T2}*/Shh*^{f} mice: mispatterned *rugae palatinae* (rugal furcations and fusions, and supernumerary *rugae*) [86, this study].

RA signaling	*Aldh1a3*: strong and persistent in HFs, and *Aldh1a1*: weak and temporally-restricted in HFs [107]. *Cyp26b1*: M associated with HFs, dermis, E of HFs, and hair placodes [69]. *RARg*/RARγ: epidermis and HFs [50]. Several components of the RA signaling pathway are expressed in sebaceous glands, the HF stem cell niche and cycling HFs [175]. *Aldh1a1*/RALDH1: TBs, LE, E of LGs and SGs, and palatal E and M (restricted) [40,107, this study] *Aldh1a2*/RALDH2: LE, E of LGs, E and M of SGs, and palatal E and M (restricted) [40,107, this study]. RALDH3: LE, E of LGs, and palatal E and M (restricted) [40, this study]. *Cyp26a1*: LE and palatal E [40,56, this sudy]. *Cyp26b1*: LM and palatal M [77, this study]. *Cyp26c1*: LE [40] and subsets of cells in *rugae palatinae* [this study]. *RARa*: E and M of SGs [78]. *RARb*: M of SGs [78]. *RARg*/RARγ: TBs, LE, LM, palatal E and M, E of SGs, and E of LGs [40,50,78, this study].	- *Cyp26b1*$^{n/n}$ mice: developmental arrest of HFs and abnormal epidermal differentiation [176].
Pharmacological enhancement of RA signaling	NA	- *In vitro* exposure of embryonic skin to vitA or RA (mice): glandular metaplasia (complete or partial transformation of whisker follicles into sero-mucous glands resembling immature SGs) [177,178].<br>- *In vitro* exposure of embryonic skin to RA or RAR agonists (mice): glandular metaplasia (transformation of whisker follicles into glands) [179].<br>- Overexposure of embryonic epidermis to vitA (chick): glandular metaplasia (transformation of feather follicles and epidermis into glands resembling nasal mucous glands) [180].<br>- *In vitro* or *in vivo* exposure of cheek pouch to retinoids (hamsters): glandular metaplasia (transformation of pouch E into glands resembling SGs) [181-183].<br>- *In vitro* exposure of embryonic tongue to RA or RAR agonists (mice): glandular metaplasia (transformation of the LE into minor SGs) [40].<br>- Overexposure to exogenous RA (rats): mispatterned *rugae palatinae* (rugal furcations and fusions, and supernumerary *rugae*) [94].<br>- Exposure of *Ptch*$^{n/+}$ mice to the retinoid tazarotene: inhibition of basal cell carcinoma development [184].
**Limb development**		
Hedgehog signaling	*Shh*: ZPA [185,186]. *Ptch1* and *Gli1*: posterior two thirds of the limb [186]. *Ptch1*: AER [187]. GLI3 activity: required to prevent *Shh* expression in the anterior region of limb buds [1]. *Ihh*: CMC, prehypertrophic and early hypertrophic chondrocytes, *Ptch1* and *Gli1*: IHH-responding cells [1,72].	- *Shh*$^{n/n}$ mice: oligodactyly (agenesis of digits in FLs, and formation of one digit in HLs) [1,44,186], unrecognizable radius and ulna, and absent or severely shortened and malformed tibia and fibula [1,44].<br>- *ShhGFPCre/Shh*^{f} mice: oligodactyly [188].<br>- *Prx1Cre/Shh*$^{f/f}$ mice: oligodactyly [188].<br>- *Msx2-Cre/Smo*$^{f/f}$ mice: supernumerary postaxial digit-like cartilage condensations in FL and HL [187], and increased length of digits 3 and 5 [189].<br>- *Gli2*^{zfd} mice: severely shortened stylopod (humerus and femur) and zeugopod (radius, ulna, tibia and fibula) of FL and HL [146].<br>- Exposure of limb buds to cyclopamine (chick): several limb anomalies, including shortening, fusion and/or reduction of limb skeletal elements the severity of which depends on the stage of exposure to cyclopamine [188].
RA signaling	*Aldh1a2*/RALDH2: LPM (E8.25-E9), presomitic and somitic mesoderm (E9-E12.5), and perichondrium [48,49,132,133]. *Rdh10*: LPM and somites [135]. *Cyp26a1*: limb bud ectoderm (transient expression) and CMC [55,56]. *Cyp26b1*: distal M of limb buds [26,57,68], CMC, perichondrium and tendons [26,69]. *RARa*: E and M of limb bud, *RARb*: proximal M of limb bud, and interdigital tissue, and *RARg*: limb bud M and CMC [78].	- *Cyp26b1*$^{n/n}$ mice: oligodactyly, shortening of stylopod and zeugopod in FL and HL, fusion of stylopod and zeugopod in FL and HL [26].

Pharmacological enhancement of RA signaling	NA	- Exposure of wing buds to RA (chick): induction of *Shh* expression, and wing duplication [1,186].<br>- Overexposure to exogenous RA (mice): phenocopies the limb skeletal defects of *Cy26b1*$^{n/n}$ mice [26], absence of femur and fibula [65], and severe truncation of all long bones of FL due to enhanced apoptosis in the limb mesenchyme [190].<br>- Overexposure to exogenous RA at E4.5-E5.5 (mice): limb duplications [143].<br>- Overexposure to vitA (mice): severe shortening or agenesis of zeugopods and stylopods of FL and HL, agenesis or severe shortening of digit 2 of FL and HL, and syndactyly [142].<br>- Overexposure to exogenous RA (hamsters): severe shortening of FL and HL, and agenesis of digits in FL and HL [61].<br>- Exposure to RAR agonists (mice): FL and HL phocomelia and oligodactyly/ectrodactyly [64].

Abbreviations: AER, apical ectodermal ridge; *Aldh1a1-3*, genes encoding the retinaldehyde dehydrogenases RALDH1-3; BA, branchial arch; BA1, first branchial arch; *Boc*, brother of CDO; *Cdo*, Cell adhesion associated oncogene regulated; CMC, chondrogenic mesenchymal condensations (chondrogenic precursors); *Cyp26a1*, *Cyp26b1*, and *Cyp26c1*, genes encoding cytochrome-P450 enzymes responsible for RA catabolism; *Dhh*, *Desert hedgehog*; E, epithelium; f, floxed allele; FL, forelimb; FNP, frontonasal process; *Gas1*, Growth-arrest sepecific1; HFs, hair follicles; Hh, Hedgehog; HL, hindlimb; HPE, holoprosencephaly; GIT, gastrointestinal tract; GT, genital tubercle; *Ihh*/IHH, Indian hedgehog gene/protein; IP, incomplete penetrance of the phenotype; LE, lingual epithelium; LG, lingual posterior glands; LM, lingual mesenchyme; LPM, lateral plate mesoderm; LR asymmetry, left-right asymmetry; M, mesenchyme; n, null allele; NA, not applicable, NCC, neural crest cells; NTD (neural tube defect *i.e.*, non-closure of the neural tube); OV, otic vesicle; P0, at birth; r, rhombomere; RA, *all-trans*-retinoic acid; RAR, retinoic acid receptor; RPE, retinal pigment epithelium; RXR, retinoid X receptor; *Shh*/SHH, Sonic hedgehog gene/protein; *Smo*, *Smoothened*; SG, salivary glands; SMMCI, solitary median maxillary central incisor; TBs, taste buds; TAM, tamoxifen; vitA, vitamin A; VAD, vitamin A-deficient; ZPA, zone of polarizing activity. The text in blue color describes similar anomalies caused by loss of Hedgehog signaling or overactivation of RA signaling.
